# Supplementary material for: Intrinsic Cellular Susceptibility to Barrett’s Esophagus in Adults Born with Esophageal Atresia
Source: Cancers (Basel). 2022 Jan 20;14(3):513. doi: 10.3390/cancers14030513 (PMC8833471; doi:10.3390/cancers14030513)
Supplement: Supplementary file 1 [file cancers-14-00513-s001.zip › cancers-1517820-supplementary/supplementary/cancers-1517820-supplementary.pdf]

cancers

MDPI

Article

Intrinsic Cellular Susceptibility to Barrett's Esophagus in Adults Born with Esophageal Atresia

Chantal A. ten Kate [1,2,3], Annelies de Klein [3], Bianca M. de Graaf [3], Michail Doukas [4], Antti Koivusalo [5], Mikko P. Pakarinen [5], Robert van der Helm [3], Tom Brands [3], Hanneke IJsselstijn [1], Yolande van Bever [3], René M.H. Wijnen [1], Manon C.W. Spaander [2] and Erwin Brosens [3,*]

Supplementary Material Content

Supplementary Tables and Figures:

Table S1: Genes and polymorphisms associated with Barrett's esophagus (BE), with selected proxy SNPs used for SNP array genotyping

Table S2: Genes and polymorphisms associated with Barrett's esophagus (BE), esophageal adenocarcinoma (EAC) or esophageal squamous cell carcinoma (ESCC)

Table S3: Odds ratios calculated from the single nucleotide polymorphism (SNP) genotyping data

Table S4: Overview of polygenic risk scores (PGRS) for all groups

Table S5: Overview of the selected odds ratios (OR) used for the polygenic risk score

Table S6: Comparison of all groups separately for the polygenic risk score (PGRS)

Table S7: Overview of survival rates of fibroblast cells after exposure to pH adjusted medium

Table S8: Basic characteristics of selected patients and controls for RNA sequencing of the esophageal biopsy specimen and for the SNP array genotyping

Table S9: Phenotype description

Table S10: Summarized results of reassessments of pathology slides of esophageal biopsy specimens

Table S11: Complete results of reassessments of pathology slides of esophageal biopsy specimens

Table S12: Results of RNA and DNA isolation

Table S13: Quality report of RNA sequencing data from esophageal biopsy specimens

Table S14: Results of RNA isolation from fibroblast of the acid exposure experiments

Table S15: Number of significantly differently expressed genes when comparing the different subgroups

Table S16: Canonical pathways, significantly enriched by differentially expressed genes, and corresponding diseases and bio functions

Table S17: Canonical pathways, enriched by differentially expressed genes

Table S18: Canonical pathways, significantly enriched by differentially expressed genes

Figure S1: Overview of survival rates of fibroblast cells after exposure to pH adjusted medium.

Figure S2: Examples of the review-based checklist used for the histopathological assessments

Figure S3: Two-dimensional scatter plot of principal component analysis (PCA)

Figure S4: Tree-dimensional scatter plot of principal component analysis (PCA)

Figure S5: Morphology (20x magnification) of fibroblast cells

Figure S6: Heat map of mean transcript per million (TPM) for fibroblast cells after the in vitro experiment

Figure S7: Number of differentially expressed genes between patients with EA and healthy controls

Figure S8: Violin plots of gastroesophageal (GEJ) samples and box plots of acid-exposed fibroblasts
Figure S9. SPINK1 is a potential BE biomarker
Datafile S1: Individual gene counts (Excel sheet)

Supplementary Methods:

SM1: Sample extraction protocol and storage
SM2: RNA and DNA isolation
SM3: SNP genotyping
SM4: RNA sequencing
SM5: Acid exposure experiments.

Table S1. Genes and polymorphisms associated with Barrett's esophagus (BE), with selected proxy SNPs used for SNP array genotyping.

Gene	SNP	Position hg19	Risk Allele	Associated with	OR (95% CI) ± SE (β)	Type of Study	Number of Patients (n)	Number of Controls (n)	Ref	Proxy SNP	D′	R′
ALDH1A2	rs3784262	chr15:58253106	C	BE, EAC	0.90 (0.87-0.93)	Meta-analysis	10038	27975	[55]	rs3204689	0.9677	0.9214
BARX1 *	rs11789015	chr9:96716028	G	BE	0.86 (0.81-0.92)	Meta-analysis	5027	15289	[55]	rs11789015	1	1
					0.85 (0.79-0.91)	GWAS	3175	10117	[56]			
CCND1	rs9344	chr11:69462910	A	BE	1.40 (0.76-2.56) [A]	Case-control	125	95	[57]	rs9344	1	1
CDX1	rs717746	chr5:149556558	G	BE	2.07 (1.05-4.08) [B]	Case-control	109	223 [E]	[58]	rs717746	1	1
CDX2	rs4769585	chr13:28550578	C	BE	2.68 (1.20-5.98) [C]	Case-control	109	223 [E]	[58]	rs6491244	0.9880	0.9722
CRTC1	rs10423674	chr19:18817903	T	BE	0.89 (0.95-0.93)	Meta-analysis	6605	23888	[55]	rs10423674	1	1
					0.85 (0.80-0.91)	GWAS	3175	10117	[56]			
FOXF1 *	rs9936833	chr16:86403118	C	BE	1.14 (1.10-1.19)	GWAS	7838	17997	[59]	rs9936833	1	1
					1.13 (0.98-1.29)	Case-control	1065	1019	[60]			
FOXP1 *	rs2687201	chr3:70928930	A	BE	1.16 (1.10-1.23)	Meta-analysis	5027	15289	[55]	rs287201	1	1
					1.18 (1.10-1.26)	GWAS	3175	10117	[56]			
GDF7 *	rs3072	chr2:20878406	G	BE	1.14 (1.09-1.18)	GWAS	10158	21062	[55]	rs9306894	0.9914	0.9662
GSTP1	rs1695	chr11: 67352689	G	BE	2.56 (1.30-5.05)	Case-control	22	173	[61]	rs1695	1	1
					1.50 (1.16-1.95)	Meta-analysis	434	738	[62]			
IGF1	rs6214	chr12:102793569	A	BE	0.90 (0.59-1.37) [D]	Case-control	207	244	[63]	rs6214	1	1
ILI12B	rs3212227	c:158742950	C	BE	1.82 (1.17-2.69)	Case-control	255	247 [F]	[64]	rs3213094	1	1
KHDRBS2-MTRNR2L9	rs62423175	chr6:62195368	A	BE	1.14 ± 0.03	Meta-analysis	6167	17159	[65]	rs1516709	0.9149	0.6837
LINC00208-BLK	rs10108511	chr8:11435516	T	BE	1.14 ± 0.02	Meta-analysis	6167	17159	[65]	rs2898290	0.9959	0.9565
MGST1	rs7312090	chr12:16515945	T	BE	1.16 (1.07-1.25)	Case-control	3288	3203	[66]	rs6488840	1	0.9928
	rs4149186	chr12:16498700	C	BE	1.11 (1.02-1.21)	Case-control	3288	3203	[66]	rs7312090	1	1
MHC region	rs9257809	chr6:29356331	A	BE	1.12 (1.13-1.28)	GWAS	7838	17997	[59]	rs9257809	1	1
				BE	1.26 ± 0.04	Meta-analysis	6167	17159	[11]			
MSRA	rs17749155	chr8:10068073	A	BE	1.20 ± 0.03	Meta-analysis	6167	17159	[11]	rs7832976	0.9829	0.9045
SATB2	rs139606545	chr2:200045039	T	BE	0.91 ± 0.02	Meta-analysis	6167	17159	[11]	rs4675343	0.9958	0.9188
TBX5 *	rs2701108	chr12:114674261	C	BE	0.90 (0.86-0.93)	GWAS	10158	21062	[55]	rs2701108	1	1
TMOD1	rs7852462	chr9:100310501	T	BE	0.87 ± 0.02	Meta-analysis	6167	17159	[11]	rs10759765	0.9225	0.824

OR = odds ratio, CI = confidence interval, SE = standardized error. * = Genes involved in foregut development. All odds ratios (OR) are presented for heterozygote risk alleles. [A] Homozygote A/A: OR 3.69 (1.46–9.29), [B] Homozygote G/G: OR 3.65 (1.73–7.69), [C] Homozygote C/C: OR 2.56 (1.10-

5.94), [D] Homozygote A/A: OR 0.43 (0.24–0.75). [E] Controls were patients with gastroesophageal reflux disease, defined as either endoscopic erosive esophagitis or complaints of substernal chest burning and/or regurgitation. [F] Controls were patients with reflux esophagitis.

Table S2. Genes and polymorphisms associated with Barrett's esophagus (BE), esophageal adenocarcinoma (EAC) or esophageal squamous cell carcinoma (ESCC).

Gene	SNP	Position hg19	Risk Allele	Associated with	OR (95% CI) ± SE (β)	Type of Study	Number of Patients (n)	Number of Controls (n)	Ref
ABCC5-HTR3c	rs9823696	chr3:183783353	A	EAC	1.17 (1.11-1.24)	Meta-analysis	4112	17159	[11]
BARX1 *	rs11789015	chr9:96716028	G	BE, EAC	0.85 (0.81-0.89)	Meta-analysis	4242	15292	[55]
				EAC	0.81 (0.75-0.88)	GWAS	2390	10120	[56]
				EAC	0.87 (0.75-1.02)	Case-control	1065	1019	[60]
				ESCC	0.77 (0.65-0.90)	Case-control	2119	2463	[66]
CAMTA1	rs17030152	chr1:7083719	C	EAC	0.87 (0.75-1.02)	Case-control	1065	1019	[60]
CCND1	rs9344	chr11:69462910	A	EAC [A]	1.37 (0.57-3.26)	Case-control	56	95	[57]
CDX1	rs3776083	chr5:149567970	A	BE	1.47 (0.61-3.54)	Case-control	109	223 [B]	[58]
CDX2	rs3812863	chr13:28545268	A	BE	1.95 (0.89-4.24)	Case-control	109	223 [B]	[58]
CHEK2	rs738722	chr22:28130012	T	ESCC	1.30 (1.19-1.43)	GWAS	2115	3202	[67]
CFTR	rs17451754	chr7:117256712	A	BE	0.87 ± 0.03	Meta-analysis	6167	17159	[11]
				EAC	0.80 ± 0.04	Meta-analysis	4112	17159	[11]
CRTC1	rs10419226	chr19:18803172	A	BE	1.19 (1.12-1.26)	GWAS	3175	10117	[56]
	rs199620551	chr19:18804295	T	BE	0.90 ± 0.02	Meta-analysis	6167	17159	[11]
				EAC	0.90 ±0.03	Meta-analysis	4112	17159	[11]
				EAC	0.85 (0.79-0.91)	GWAS	2390	10120	[56]
DIO3	rs2895917	chr14:102052775	T	EAC	0.88 (0.76-1.02)	Case-control	1065	1019	[60]
FOXF1	rs2178146	chr16:86463695	G	BE	0.89 (0.84-0.95)	GWAS	3175	10117	[56]
	rs3111601	chr16:86400081	G	BE	1.13 (1.05-1.20)	GWAS	3175	10117	[56]
				EAC	1.16 (1.08-1.24)	GWAS	2390	10120	[56]
	rs2178146	chr16:86463695	G	EAC	0.85 (0.79-0.91)	GWAS	2390	10120	[56]
	rs9936833	chr16:86403118	C	EAC	1.21 (0.99-1.47)	Case-control	318	605	[68]
FOXF1-LOC732275 *	rs1979654	chr3:86396835	C	BE	0.90 ± 0.02	Meta-analysis	6167	17159	[11]
				EAC	0.90 ± 0.03	Meta-analysis	4112	17159	[11]
FOXP1 *	rs2687202	chr3:70929983	T	BE	1.13 ± 0.02	Meta-analysis	6167	17159	[11]
				EAC	1.13 ± 0.03	Meta-analysis	4112	17159	[11]
	rs9837992	chr3:70959438	A	EAC	1.23 (1.07-1.42)	Case-control	1065	1019	[60]
FOXP1 *	rs2687201	chr3:70928930	A	BE, EAC	1.17 (1.11-1.23)	Meta-analysis	4242	15292	[55]

				EAC	1.20 (1.12-1.29)	GWAS	2390	10120	[56]
				EAC	1.26 (1.09-1.46)	Case-control	1065	1019	[60]
GATA6	rs4800353	chr18:19654137	G	EAC	0.83 (0.69-1.01)	Case-control	1065	1019	[60]
*GDF7-LDAH**	rs7255	chr2:20878820	C	BE	1.12 ± 0.02	Meta-analysis	6167	17159	[11]
				EAC	1.17 ± 0.03	Meta-analysis	4112	17159	[11]
GHR	rs6898743	chr5:42602492	G	EAC	0.42 (0.23-0.76)	Case-control	210	240	[63]
GSTP1	rs1695	chr11: 67352689	G	EAC	1.73 (0.75-4.02)	Case-control	12	21	[69]
				EAC	1.20 (0.94-1.54)	Meta-analysis	432	1086	[62]
KHDRBS2-MTRNR2L9	rs62423175	chr6:62195368	A	EAC	1.23 ± 0.0377	Meta-analysis	4112	17159	[11]
LINC00208-BLK	rs10108511	chr8:11435516	T	EAC	1.08 ± 0.03	Meta-analysis	4112	17159	[11]
MFHAS1	rs4523255	chr8:8713038	T	EAC	1.14 (0.99-1.31)	Case-control	n = 1065	n = 1019	[60]
MGST1	rs4149203	chr12:16514921	T	BE	1.16 (1.08-1.26	Case-control	n = 3295	n = 3207	[65]
	rs3852575	chr12:16516260	T	BE	1.16 (1.08-1.25)	Case-control	n = 3295	n = 3207	[65]
	rs1419204	chr12:16515062	C	BE	1.16 (1.08-1.25)	Case-control	n = 3295	n = 3207	[65]
	rs4149207	chr12:16517491	T	BE	1.14 (1.06-1.23)	Case-control	n = 3295	n = 3207	[65]
	rs4149208	chr12:16517581	T	BE	1.14 (1.06-1.23)	Case-control	n = 3295	n = 3207	[65]
	rs3759207	chr12:16516710	C	BE	1.14 (1.05-1.23)	Case-control	n = 3295	n = 3207	[65]
	rs4149195	chr12:16512128	G	BE	1.20 (1.07-1.35)	Case-control	n = 3295	n = 3207	[65]
	rs2239676	chr12:16500448	G	BE	1.19 (1.06-1.34)	Case-control	n = 3295	n = 3207	[65]
	rs4149187	chr12:16500071	G	BE	1.18 (1.05-1.32)	Case-control	n = 3295	n = 3207	[65]
	rs2239677	chr12:16500680	A	BE	1.38 (1.09-1.75)	Case-control	n = 3295	n = 3207	[65]
	rs2239675	chr12:16500265	G	BE	1.12 (1.02-1.23)	Case-control	n = 3295	n = 3207	[65]
	rs2975138	chr12:16501551	A	BE	1.10 (1.02-1.20)	Case-control	n = 3295	n = 3207	[65]
MHC region	rs9257809	chr6:29356331	G	EAC	1.14 ± 0.05	Meta-analysis	4112	17159	[11]
				ESCC	1.76 (1.16-2.66)	Case-control	n = 107	n = 605	[68]
MSRA	rs17749155	chr8:10068073	A	EAC	1.13 ± 0.04	Meta-analysis	4112	17159	[11]
PCDH20	rs2669333	chr13:63574196	A	EAC	1.15 (1.00-1.33)	Case-control	n = 1065	n = 1019	[60]
PLCE1	rs2274223	chr10:96066314	G	ESCC	1.34 (1.22-1.48)	GWAS	N = 2115	n = 3202	[67]
	rs3765524	chr10:96058298	T	ESCC	1.35 (1.22-1.49)	GWAS	N = 2115	n = 3202	[67]
	rs3781264	chr10:96070375	C	ESCC	1.38 (1.23-1.53)	GWAS	N = 2115	n = 3202	[67]
	rs11187842	chr10:96052511	T	ESCC	1.37 (1.23-1.53)	GWAS	N = 2115	n = 3202	[67]
	rs753724	chr10:96051417	T	ESCC	1.38 (1.23-1.54)	GWAS	N = 2115	n = 3202	[67]
SATB2	rs139606545	chr2:200045039	T	EAC	0.88 ± 0.03	Meta-analysis	4112	17159	[11]
*TBX5-LOC105369996 **	rs1247942	chr12:114673723	C	BE	0.88 ± 0.02	Meta-analysis	6167	17159	[11]
				EAC	0.90 ± 0.03	Meta-analysis	4112	17159	[11]

TMOD1	rs7852462	chr9:100310501	T	EAC	0.93 ± 0.03	Meta-analysis	4112	17159	[11]
TPPP-CEP72	rs9918259	chr5:663092	T	BE	1.20 ± 0.04	Meta-analysis	6167	17159	[11]
				EAC	1.20 ± 0.04	Meta-analysis	4112	17159	[11]
XRCC2	rs11771429	chr7:153271877	T	EAC	0.85 (0.71-1.02)	Case-control	n = 1065	n = 1019	[60]

These polymorphisms were not selected for SNP array genotyping because no proxy SNP could be selected, or because they were only associated with EAC or ESCC and not with BE. OR = odds ratio, CI = confidence interval, SE = standardized error. * = Genes involved in foregut development. All odds ratios (OR) are presented for heterozygote risk alleles. [A] Homozygote A/A: OR 5.99 (1.86–18.96). [B] Controls were patients with gastroesophageal reflux disease, defined as either endoscopic erosive esophagitis or complaints of substernal chest burning and/or regurgitation.

Table S3. Odds ratios calculated from the single nucleotide polymorphism (SNP) genotyping data.

		**Group A (EA/BE) vs. Controls**		**Group B (EA only) vs. Controls**		**Group C (BE only) vs. Controls**		**Group A (EA/BE) vs. Group C (BE only)**	
**Gene**	**SNP**	***OR (95% CI)***	***p-Value X^2***	***OR (95% CI)***	***p-Value X^2***	***OR (95% CI)***	***p-Value X^2***	***OR (95% CI)***	***p-Value X^2***
ALDH1A2	rs3784262	2.28 (1.08-4.83)	**0.028** *	0.53 (0.22-0.83)	**0.005** *	0.58 (0.24-1.40)	0.222	3.94 (1.24-12.4)	**0.017** *
BARX1	rs11789015	1.70 (0.87-3.31)	0.116	1.43 (0.91-2.26)	0.124	0.97 (0.35-2.68)	0.956	1.75 (0.53-5.82)	0.361
CCND1	rs9344	1.04 (0.54-1.97)	0.916	0.80 (0.52-1.23)	0.306	1.41 (0.58-3.40)	0.450	0.74 (0.25-2.17)	0.581
CDX1	rs717746	1.14 (0.60-2.16)	0.695	1.72 (1.11-2.67)	**0.014** *	0.61 (0.24-1.54)	0.294	1.86 (0.61-5.65)	0.275
CDX2	rs4769585	1.28 (0.66-2.46)	0.463	1.05 (0.68-1.61)	0.832	0.45 (0.18-1.13)	0.082	2.85 (0.93-8.73)	0.064
CRTC1	rs10423674	1.14 (0.59-2.22)	0.693	0.58 (0.35-0.96)	**0.033** *	1.06 (0.42-2.65)	0.908	1.08 (0.35-3.34)	0.890
FOXF1	rs9936833	0.56 (0.27-1.16)	0.116	0.81 (0.52-1.28)	0.368	0.52 (0.19-1.44)	0.205	1.07 (0.31-3.69)	0.913
FOXP1	rs2687201	1.41 (0.73-2.72)	0.306	0.86 (0.53-1.38)	0.526	4.01 (1.60-10.07)	**0.002** *	0.35 (0.11-1.08)	0.064
GDF7	rs3072	0.45 (0.21-0.99)	0.044	0.93 (0.59-1.45)	0.740	2.08 (0.86-5.03)	0.098	0.22 (0.07-0.70)	**0.009** *
GSTP1	rs1695	1.57 (0.82-2.99)	0.170	0.81 (0.51-1.30)	0.387	0.83 (0.32-2.16)	0.705	1.89 (0.60-5.93)	0.276
IGF1	rs6214	0.48 (0.23-0.98)	**0.041** *	0.76 (0.49-1.18)	0.225	0.80 (0.35-1.83)	0.593	0.60 (0.20-1.77)	0.352
ILI12B	rs3212227	1.21 (0.57-2.56)	0.629	0.67 (0.37-1.23)	0.195	1.29 (0.47-3.57)	0.619	0.93 (0.27-3.26)	0.911
KHDRBS2-MTRNR2L9	rs62423175	1.40 (0.69-2.84)	0.352	0.76 (0.44-1.33)	0.341	1.14 (0.42-3.16)	0.793	1.22 (0.36-4.16)	0.749
LINC00208-BLK	rs10108511	1.31 (0.69-2.49)	0.415	1.06 (0.69-1.63)	0.793	1.06 (0.44-2.55)	0.898	1.24 (0.42-3.64)	0.703
MGST1	rs4149186	1.51 (0.76-3.02)	0.238	1.30 (0.81-2.10)	0.279	1.09 (0.40-3.02)	0.863	1.38 (0.41-4.66)	0.601
MGST1	rs7312090	0.57 (0.26-1.25)	0.158	1.00 (0.63-1.58)	0.995	0.54 (0.18-1.60)	0.258	1.07 (0.28-4.07)	0.925
MHC region	rs9257809	0.67 (0.26-1.73)	0.404	0.89 (0.44-1.80)	0.741	0.91 (0.21-3.93)	0.890	0.73 (0.13-4.13)	0.726
MSRA	rs17749155	0.51 (0.18-1.43)	0.194	0.82 (0.46-1.46)	0.494	0.11 (0.36-3.23)	0.895	0.47 (0.11-2.11)	0.320
SATB2	rs139606545	0.73 (0.37-1.41)	0.347	0.75 (0.48-1.16)	0.198	0.42 (0.15-1.14)	0.081	1.75 (0.53-5.82)	0.361
TBX5	rs2701108	1.45 (0.76-2.79)	0.260	1.45 (0.94-2.24)	0.094	0.67 (0.24-1.83)	0.431	2.18 (0.66-7.20)	0.198
TMOD1	rs7852462	0.96 (0.50-1.83)	0.898	0.96 (0.62-1.47)	0.839	1.08 (0.45-2.61)	0.868	0.89 (0.30-2.63)	0.832

Group A = patients with esophageal atresia (EA) and Barrett's esophagus (BE) (n = 19); Group B = patients with EA without BE (n = 44); Group C = patients with BE without EA in history (n = 10); controls n = 730. OR = odds ratio, CI = confidence interval, X^2 = chi-square test. Asterisk indicates significance level $p < 0.05$.

Table S4. Overview of polygenic risk scores (PGRS) for all groups, based on odds ratios (ORs) selected from the literature (left) and ORs calculated from the SNP array (right).

Group	n	PGRS Based on ORs from Literature			PGRS Based on ORs Calculated from Our Study Population		
		Median (range)	*IQR*	*Kruskal-Wallis Test*	*Median (range)*	*IQR*	*Kruskal-Wallis Test*
Group A (EA/BE)	19	3.24 (1.39-4.68)	1.40	0.495	3.05 (0.14-6.04)	1.70	**0.001** *
Group B (EA only)	44	2.98 (1.19-4.74)	1.28		2.52 (-2.73-5.72)	3.38	
Group C (BE only)	10	2.63 (1.85-3.53)	1.17		-0.24 (-2.83-2.15)	2.42	
Controls	730	3.00 (-0.28-5.78)	1.65		2.21 (-4.44-7.83)	2.21	

Group A = patients with esophageal atresia (EA) and Barrett's esophagus (BE); group B = patients with EA without BE; and group C = patients with BE without EA in history. IQR = interquartile range. Asterisk indicates significance level $p < 0.05$.

Table S5. Overview of the selected odds ratios (OR) used for the polygenic risk score.

Gene	SNP	Proxy SNP	Literature *OR (95% CI) ± SE (β)*	SNP array Data (n = 29) *OR (95% CI)*
ALDH1A2	rs3784262	rs3204689	0.90 (0.87-0.93) [55]	3.94 (1.24-12.4)
BARX1	rs11789015	rs11789015	0.86 (0.81-0.92) [55]	1.75 (0.53-5.82)
CCND1	rs9344	rs9344	1.40 (0.76-2.56) [57]	0.74 (0.25-2.17)
CDX1	rs717746	rs717746	2.07 (1.05-4.08) [58]	1.86 (0.61-5.65)
CDX2	rs4769585	rs6491244	2.68 (1.20-5.98) [58]	2.85 (0.93-8.73)
CRTC1	rs10423674	rs10423674	0.89 (0.95-0.93) [55]	1.08 (0.35-3.34)
FOXF1	rs9936833	rs9936833	1.14 (1.10-1.19) [59]	1.07 (0.31-3.69)
FOXP1	rs2687201	rs2687201	1.16 (1.10-1.23) [55]	0.35 (0.11-1.08)
GDF7	rs3072	rs9306894	1.14 (1.09-1.18) [55]	0.22 (0.07-0.70)
GSTP1	rs1695	rs1695	1.50 (1.16-1.95) [62]	1.89 (0.60-5.93)
IGF1	rs6214	rs6214	0.90 (0.59-1.37) [63]	0.60 (0.20-1.77)
ILI12B	rs3212227	rs3213094	1.82 (1.17-2.69) [64]	0.93 (0.27-3.26)
KHDRBS2-MTRNR2L9	rs62423175	rs1516709	1.14 ± 0.03 [11]	1.22 (0.36-4.16)
LINC00208-BLK	rs10108511	rs2898290	1.14 ± 0.02 [11]	1.24 (0.42-3.64)
MGST1	rs4149186	rs6488840	1.11 (1.02-1.21) [65]	1.38 (0.41-4.66)
MGST1	rs7312090	rs7312090	1.16 (1.07-1.25) [65]	1.07 (0.28-4.07)
MHC region	rs9257809	rs9257809	1.26 ± 0.04 [11]	0.73 (0. 13-4.13)
MSRA	rs17749155	rs7832976	1.20 ± 0.03 [11]	0.47 (0.11-2.11)
SATB2	rs139606545	rs4675343	0.91 ± 0.02 [11]	1.75 (0.53-5.82)
TBX5	rs2701108	rs2701108	0.90 (0.86-0.93) [65]	2.18 (0.66-7.20)
TMOD1	rs7852462	rs10759765	0.87 ± 0.02 [11]	0.89 (0.30-2.63)

In case multiple studies published an OR for a certain SNP, the study with the largest sample size was included in the PGRS. For *MGST1* two SNPs were described, not in linkage disequilibrium with each other, for which both SNPs were included. OR = odds ratio, CI = confidence interval, SE = standardized error.

Table S6. Comparison of all groups separately for the polygenic risk score (PGRS) based on odds ratios (ORs) selected from the literature (left) and ORs calculated from the SNP array (right), using Mann-Whitney tests.

Group	PGRS based on ORs from literature<br>*p-value*	PGRS based on ORs calculated from our study population<br>*p-value*
Group A (EA/BE) vs. Group B (EA only)	0.500	0.274
Group A (EA/BE) vs. Group C (BE only)	0.069	**<0.001 ***
Group A (EA/BE) vs. Controls	0.381	0.055
Group B (EA only) vs. Group C (BE only)	0.124	**0.001 ***
Group B (EA only) vs. Controls	0.694	0.568
Group C (BE only) vs. Controls	0.251	**<0.001 ***

Group A = patients with esophageal atresia (EA) and Barrett's esophagus (BE) (n = 19); Group B = patients with EA without BE (n = 44); Group C = patients with BE without EA in history (n = 10); controls n = 730.

Table S7. Overview of survival rates of fibroblast cells after exposure to pH adjusted medium.

**Experiment A**		**Experiment B**		
**Exposure * (pH)**	**Survival (%)**	**Exposure (pH – minutes)**	**Survival (%)**	
	Controls		*Patients*	*Controls*
1.46	43.9	1.47 – 30	48.8	53.8
1.99	50.9	1.47 – 60	52.3	51.5
2.38	49.7	1.47 – 120	55.1	55.2
3.31	56.7	3.46 – 30	50.4	54.5
3.49	62.0	3.46 – 30	50.7	51.6
		3.46 – 30	50.0	48.7
		7.70 - 30	80.0	76.7

* = 30 minutes. Experiment A are the pooled results of a duplo experiment on three control cell lines. Experiment B are the pooled results of a duplo experiment on three patient cell lines and three control cell lines.

Table S8. Basic characteristics of selected patients and controls for RNA sequencing of the esophageal biopsy specimen (upper), and for the SNP array genotyping (under).

		**Group A (EA/BE, n = 11)**	**Group B (EA only, n = 10)**		**Group C (BE only, n = 10)**	
				p-value ^{A}		*p-value* ^{B}
**Selected patients for RNA sequencing**	**Male (%)**	10 (90.9)	6 (60.0)	0.114	7 (70.0)	0.221
	**Type of EA** ^{C}					
	Type A	1 (9.1)	0	0.563		
	Type C	8 (72.7)	6 (60.0)	0.525		
	Type E	0	1 (10.0)	0.437		
	Unknown	2 (18.2)	3 (30.0)	N/E		
	**Staged repair**	1 (9.1)	0 (0%)	0.242		
	**Fundoplication surgery in history**	4 (36.4)	2 (20.0)	0.274	1 (10.0)	0.162
	**Median age at time of biopsy (range)**	39.3 (20.5-58.7)	29.4 (21.8-49.3)	0.099	59.1 (45.2-66.7)	**0.003 ***
	**Tobacco smoking**					
	No	5 (45.5)	7 (70.0)	0.189	6 (60.0)	0.231
	Former smoker	4 (36.4)	1 (10.0)	0.162	2 (20.0)	0.307
	Yes, active smoker	2 (18.2)	2 (20.0)	0.414	1 (10.0)	0.434
	Missing	0	0	N/E	1 (10.0)	N/E

	**Alcohol consumption**					
	No alcohol	2 (18.2)	2 (20.0)	0.409	1 (10.0)	0.485
	≤7 units/week	8 (72.7)	8 (80.0)	0.383	4 (40.0)	0.400
	≥8 units/week	1 (9.1)	0	0.550	1 (10.0)	0.485
	Missing	0	0	N/E	4 (40.0)	N/E
	**Endoscopic esophagitis** [D]					
	No	6 (54.5)	10 (100.0)	**0.023 ***	9 (90.0)	0.085
	Grade A	4 (36.4)	0	0.055	0	0.055
	Grade B	1 (9.1)	0	0.524	1 (10.0)	0.524
	**Length of BE**					
	Short segment, <3 cm	5 (45.5)			0	**0.023 ***
	Long segment, ≥3 cm	6 (54.5)			10 (100.0)	**0.023 ***
	**Dysplasia**	0			0	

		**Group A (EA/BE, n = 19)**	**Group B (EA only, n = 44)**	**Group C (BE only, n = 10)**	**Controls (n = 730)**
**Selected patients for SNP array genotyping**	**Male (%)**	14 (73.3)	26 (59.1)	7 (70.0)	340 (46.6)
	**Type of EA**				
	Type A	1 (5.3)	2 (4.5)		
	Type C	16 (84.2)	37 (84.1)		
	Type D	0	1 (2.3)		
	Type E	0	1 (2.3)		
	Unknown	2 (10.5)	3 (6.8)		
	**Length of BE**				
	Short segment, <3 cm	9 (47.4)		0	
	Long segment, ≥3 cm	10 (52.6)		10 (100.0)	

Data is presented as n (%) or median (range). Asterisk indicates significance ($p < 0.05$). EA = esophageal atresia, BE = Barrett's esophagus, N/E = not evaluated. [A] Mann-Whitney test, group A versus group B. [B] Mann-Whitney test, group A versus group C. [C] According to the Gross classification [70]. [D] According to the Los Angeles criteria [71]. EA/BE patients were significantly younger than BE only patients (median age 39.3 versus 59.1 years, $p = 0.003$). BE only patients had more often long segment BE (≥3 cm) compared with EA/BE patients ($p = 0.023$).

Table S9. Phenotype description.

	Individual	Gender	EA type [A]	Phenotype	Remarks
**Group A (EA/BE)**	BBO-007	male	C	EA/TEF, inguinal hernia	-
	BBO-021	male	C	EA/TEF, congenital hiatal hernia	-
	BBO-027	male	C	EA/TEF, IHPS, extra ribs, fusion of vertebrae, macrocephaly, bulbar dermoid cyst, auricular tags, short thick/broad neck	Oculo-auriculo-vertebral spectrum, Klippel-Feil
	BBO-038	male	C	EA/TEF, anorectal malformation, ureteral duplication, aplasia left kidney, dysplasia right kidney, hypospadias, hip luxation	VACTERL
	BBO-053	female	unknown	EA/TEF	-
	BBO-058	male	C	EA/TEF, abnormal thoracic and lumbal vertebrae, congenital scoliosis, undescended testicles, inguinal hernia	-
	BBO-060	male	C	EA/TEF	-
	BBO-061	male	A	EA, adduction pollex	-
	BBO-063	male	C	EA/TEF	-
	BBO-074	male	unknown	EA/TEF[#]	-

	BBO-080	male	C	EA/TEF, atrial septal defect type II, perimembranous ventricular septal defect	
**Group B (EA only)**	BBO-065	male	C	EA/TEF	-
	BBO-066	male	E	TEF, hemivertebrae, fusion of vertebrae, 13 costae, anorectal malformation	-
	BBO-070	female	unknown	EA/TEF	-
	BBO-072	female	unknown	*Patient records could not be retrieved from archives*	-
	BBO-077	male	C	EA/TEF	-
	BBO-086	male	C	EA/TEF, microcephaly, microdactylia	Feingold syndrome
	BBO-087	male	C	EA/TEF, IHPS, mild left sided expansion of the pyelocaliceal system , breath holding spells	-
	BBO-090	female	C	EA/TEF, anorectal malformation (vestibular anus), atrial septal defect type II, perimembranous ventricular septal defect, hypertelorism, protruding ears, sacral dimple	VACTERL, Townes Brocks (Sall1)
	BBO-092	female	C	EA/TEF, dysmaturity	-
	BBO-094	male	unknown	EA/TEF#	-
**EA [B]**	Patient 1	male	C	EA/TEF, anorectal malformation, double outlet ventricle right, aplasia right thumb, cleft palate	VACTERL
	Patient 2	female	C	EA/TEF, anorectal malformation, duodenal atresia, aplasia sacrum, vertebral anomalies, aplasia right kidney, cleft of lip and palate,	VACTERL
	Patient 3	female	C	EA/TEF, perimembranous ventricular septal defect	-

EA = esophageal atresia, TEF = tracheoesophageal fistula, BE = Barrett's esophagus, IHPS = infantile pyloric stenosis, VACTERL = vertebral, anorectal, cardiac, tracheoesophageal, renal and limb malformations. [A] According to Gross classification [70] [B] Patients included in acid exposure experiments.

Table S10. Summarized results of reassessments of pathology slides of esophageal biopsy specimens that were included in the differential expression analysis.

	**Group A (EA/BE, n = 11)**	**Group B (EA only, n = 10)**	**Group C (BE only, n = 10)**
**Type of mucosa**			
Squamous epithelium	+	+	+
Multi-layered squamous epithelium (overlying) columnar epithelium	-	-	-
Columnar epithelium	+	+/-	+
**Gland subtype**			
Corpus glands, cardia glands or mixed	mixed	mixed [A]	mixed
**Esophagitis / GERD**			
GERD present	-	-	-
Nonspecific inflammatory cell infiltrate	+	+	+
Neutrophil granulocytes	+	-	+
**Intestinal metaplasia**			
Amount of intestinal metaplasia (average per slide)	20%	N/A	50%
Goblet cells present	+	-	+
Paneth cells present	-	+/-	+/-
**Dysplasia**			
Dysplasia present	-	-	-
Crypt architecture distorted	-	-	-
Cytology distorted	-	-	-

Plus (+) indicates the presence of a criteria in the majority of the slides. Minus (-) indicates the absence of a criteria in all slides. Plus-minus (+/-) indicate the presence of a criteria in a few slides. EA = esophageal atresia, BE = Barrett's

esophagus, GERD = gastroesophageal reflux disease, N/A = not applicable. [A] Including one patient with pseudo-pancreatic metaplasia. Columnar epithelium was present in all groups, but not in every individual patient in group B (EA only). In group B (EA only), neutrophil granulocytes were absent while a nonspecific inflammatory cell infiltrate was present in all groups. Focusing on the characteristics of BE, IM with the presence of goblet cells was similarly present in group A (EA/BE) and group C (BE only). The amount of IM was larger in group C (BE only).

Table S11. Complete results of reassessments of pathology slides of esophageal biopsy specimens that were included in the differential expression analysis.

	**Group A (EA/BE)**										
	BBO-008	BBO-021	BBO-027	BBO-038	BBO-053	BBO-058	BBO-060	BBO-061	BBO-063	BBO-074	BBO-080
**Type of mucosa**											
Squamous epithelium	+	+	+	+	+	+	+	+	+	+	+
Multi-layered squamous epithelium (overlying) columnar epithelium	-	-	-	+	-	-	-	-	-	-	-
Columnar epithelium	+	+	+	+	+	+	+	+	+	+	+
**Gland subtype**											
Corpus glands, cardia glands or mixed	M	M	M	C	M	C	M	C	Co	M	C
**Esophagitis / GERD**											
GERD present	-	-	-	-	-	-	-	-	-	-	-
Nonspecific inflammatory cell infiltrate	+	+	+	++	+	+	+	++	+	+	+
Neutrophil granulocytes	+	+	+	+	+	-	+	+	+	+	-
**Intestinal metaplasia**											
Amount of intestinal metaplasia (%)	5	20	40	5	20	70	20	5	30	5	10
Goblet cells present	+	+	+	+	+	+	+	+	+	+	+
Paneth cells present	-	+	+	-	-	-	-	-	-	-	-
**Dysplasia**											
Dysplasia present	-	-	-	-	-	-	-	-	-	-	-
Crypt architecture distorted	+	+	+	-	-	-	-	-	-	-	-
Cytology distorted											
	**Group B (EA only)**										
	BBO-065	BBO-066	BBO-070	BBO-072	BBO-077	BBO-086	BBO-087	BBO-090	BBO-092	BBO-094	
**Type of mucosa**											
Squamous epithelium	+	+	+	+	+	+	+	+	+	+	
Multi-layered squamous epithelium (overlying) columnar epithelium	-	-	-	-	-	-	-	-	-	-	
Columnar epithelium	+	-	+	+	+	+	+	+	+	-	
**Gland subtype**											
Corpus glands, cardia glands or mixed	C	N/A	M	C *	M	M	M	M *	M	N/A	
**Esophagitis / GERD**											
GERD present	-	-	-	-	-	-	+	+/-	-	-	
Nonspecific inflammatory cell infiltrate	+	-	++	+	+	+	+	++	++	-	
Neutrophil granulocytes	+	-	-	-	-	-	-	+	-	-	
**Intestinal metaplasia**	N/A	N/A	N/A	N/A	N/A	N/A	N/A	N/A	N/A	N/A	
Amount of intestinal metaplasia (%)	-	-	-	-	-	-	-	-	-	-	
Goblet cells present	-	-	-	-	-	-	-	-	-	-	
Paneth cells present											
**Dysplasia**	-	-	-	-	-	-	-	-	-	-	
Dysplasia present	-	-	-	-	-	-	-	-	-	-	
Crypt architecture distorted	-	-	-	-	-	-	-	-	-	-	

Cytology distorted	-	-	-	-	-	-	+	+/-	-	-

	**Group C (BE only)**									
	BBE-001	BBE-017	BBE-021	BBE-023	BBE-028	BBE-043	BBE-053	BBE-079	BBE-080	BBE-098
**Type of mucosa**										
Squamous epithelium	+	-	+	+	+	+	+	+	+	+
Multi-layered squamous epithelium (overlying) columnar epithelium	-	-	-	+	-	-	-	-	-	-
Columnar epithelium	+	+	+	+	+	+	+	+	+	+
**Gland subtype**										
Corpus glands, cardia glands or mixed	M	C	C	M	C	C	C	M	M	M
**Esophagitis / GERD**										
GERD present	-	-	-	-	-	-	-	-	-	-
Nonspecific inflammatory cell infiltrate	+	+	+	+	+	++	+	+	+	++
Neutrophil granulocytes	-	+	-	+	+	+	-	+	-	+
**Intestinal metaplasia**										
Amount of intestinal metaplasia (%)	60	40	65	50	15	60	70	30	45	70
Goblet cells present	+	+	+	+	+	+	+	+	+	+
Paneth cells present	+	-	-	+	+	+	+	-	+	-
**Dysplasia**										
Dysplasia present	-	-	-	-	-	-	-	-	-	-
Crypt architecture distorted	-	-	-	-	-	-	-	-	-	-
Cytology distorted	-	-	-	-	-	-	-	-	-	-

Plus (+) indicates the presence of a criteria in the majority of the slides. Minus (-) indicates the absence of a criteria in all slides. Plus-minus (+/-) indicate the presence of a criteria in a few slides. * Combined with pseudo-pancreatic metaplasia. EA = esophageal atresia, BE = Barrett's esophagus, GERD = gastroesophageal reflux disease, M = mixed glands, C = cardia glands, Co = corpus glands, N/A = not applicable.

Table S12. Results of RNA and DNA isolation from esophageal biopsy specimens, blood and skin fibroblasts in terms of quantity and quality.

	**Patient**	**RNA**						**DNA**		**Experiments that Patient Was Included in**
		**SQ**		**GEJ**		**Fibro**		**Blood**	**Fibro**	
		(ng/ul)	*RIN*	*(ng/ul)*	*RIN*	*(ng/ul)*	*RIN*	*(ng/ul)*	*(ng/ul)*	
**Group A (EA/BE)**	BBO-007	162	10	262	7.2			60.1		RNAseq of biopsy specimens, SNP genotyping
	BBO-018							57.3		SNP genotyping
	BBO-021			27	8.4			55.5		RNAseq of biopsy specimens, SNP genotyping
	BBO-027			86	8.9			56.1		RNAseq of biopsy specimens, SNP genotyping
	BBO-038	23	9.8	346	8.2			58.6		RNAseq of biopsy specimens, SNP genotyping
	BBO-053	86	9.7	341	9.1			60.1		RNAseq of biopsy specimens, SNP genotyping
	BBO-058	80	10	924	9.2			72.5		RNAseq of biopsy specimens, SNP genotyping
	BBO-060	556	9.8	699	9.1			57.0		RNAseq of biopsy specimens, SNP genotyping
	BBO-061			341	9.5			62.9		RNAseq of biopsy specimens, SNP genotyping
	BBO-063	131	10	839	9.0			61.4		RNAseq of biopsy specimens, SNP genotyping

	BBO-064					58.6		SNP genotyping
	BBO-069					59.5		SNP genotyping
	BBO-074	313	10	132	9.9	58.7		RNAseq of biopsy specimens, SNP genotyping
	BBO-080	206	9.9	388	7.6	57.3		RNAseq of biopsy specimens, SNP genotyping
	BBO-134					50.0		SNP genotyping
	BBO-142					50.0		SNP genotyping
	BBO-149					50.0		SNP genotyping
	BBO-160					50.0		SNP genotyping
	EAH1					158.3		SNP genotyping
	EAH2					111.5		SNP genotyping
	EAH3					107.2		SNP genotyping
	EAH4					151.4		SNP genotyping
	EAH5					101.5		SNP genotyping
	EAH6					86.0		SNP genotyping
	EAH7					246.0		SNP genotyping
**Group B (EA only)**	BBO-002					66.9		SNP genotyping
	BBO-003					59.9		SNP genotyping
	BBO-013					55.7		SNP genotyping
	BBO-014					56.2		SNP genotyping
	BBO-015					53.8		SNP genotyping
	BBO-020					56.3		SNP genotyping
	BBO-026					64.9		SNP genotyping
	BBO-034					72.5		SNP genotyping
	BBO-036					59.9		SNP genotyping
	BBO-050					58.1		SNP genotyping
	BBO-055					63.8		SNP genotyping
	BBO-065	34	9.6	274	10	48.0		RNAseq of biopsy specimens, SNP genotyping
	BBO-066	95	10	1035	9.9	62.9		RNAseq of biopsy specimens, SNP genotyping
	BBO-070	127	9.9	201	9.6	57.4		RNAseq of biopsy specimens, SNP genotyping
	BBO-072	165	9.9	71	9.7	55.8		RNAseq of biopsy specimens, SNP genotyping
	BBO-077	47	10	408	8.7	63.9		RNAseq of biopsy specimens, SNP genotyping
	BBO-086	125	10	385	8.1	59.4		RNAseq of biopsy specimens, SNP genotyping
	BBO-087	126	9.9	509	8.1	63.1		RNAseq of biopsy specimens, SNP genotyping
	BBO-090	99	9.6	159	9.3	54.5		RNAseq of biopsy specimens, SNP genotyping
	BBO-092			188	7.0			RNAseq of biopsy specimens
	BBO-094	62	10	617	6.7	71.9		RNAseq of biopsy specimens, SNP genotyping
	EA1						57.6	SNP genotyping
	EA2						71.8	SNP genotyping
	EA3						42.6	SNP genotyping
	EA4						42.5	SNP genotyping
	EA5						54.7	SNP genotyping
	EA6						67.3	SNP genotyping
	EA7						47.8	SNP genotyping

	EA8								43.0	SNP genotyping
	EA9								48.2	SNP genotyping
	EA10								89.2	SNP genotyping
	EA11								73.2	SNP genotyping
	EA12								69.0	SNP genotyping
	EA13								67.6	SNP genotyping
	EA14					239 / 30	9.3 / 7.3			Acid exposure experiment, including RNAseq
	EA15					206 / 70	9.3 / 9.2			Acid exposure experiment, including RNAseq
	EA16					141 / 30	9.8 / 8.3			Acid exposure experiment, including RNAseq
Group C (BE only)	BBE-001			260	8.4			39.3		RNAseq of biopsy specimens, SNP genotyping
	BBE-017	39	7.8	14 [B]	6.3			35.2		RNAseq of biopsy specimens, SNP genotyping
	BBE-021	209	9.3	756	8.3			82.3		RNAseq of biopsy specimens, SNP genotyping
	BBE-023	163	9.1	382	7.4			44.0		RNAseq of biopsy specimens, SNP genotyping
	BBE-028	71	9.1	132	7.9			41.6		RNAseq of biopsy specimens, SNP genotyping
	BBE-043	162	9.1	264	7.1			39.3		RNAseq of biopsy specimens, SNP genotyping
	BBE-053	65	9.4	112	7.5			78.3		RNAseq of biopsy specimens, SNP genotyping
	BBE-079	16 [B]	8.0	88	8.5			37.3		RNAseq of biopsy specimens, SNP genotyping
	BBE-080			376	9.4			31.0		RNAseq of biopsy specimens, SNP genotyping
	BBE-098	92	9.0	97	7.3			39.2		RNAseq of biopsy specimens, SNP genotyping
Controls	Control 1								22.0	SNP genotyping
	Control 2								47.8	SNP genotyping
	Control 3								93.2	SNP genotyping
	Control 1 [A]					31 / 13	9.0 / 7.2			Acid exposure experiment, including RNAseq
	Control 2 [A]					223 / 66	9.3 / 8.3			Acid exposure experiment, including RNAseq
	Control 3 [A]					173 / 48	9.0 / 8.1			Acid exposure experiment, including RNAseq

Group A = patients with esophageal atresia (EA) and Barrett's esophagus (BE); group B = patients with EA without BE; and group C = patients with BE without EA in history. SQ = squamous cell epithelium, GEJ = gastroesophageal junction, RIN = RNA integrity number. A The polygenetic risk score of these individuals was respectively 1.19, 3.18 and 3.26 for the patients, and 1.73, 3.30 and 4.80 for the controls. RNA amount is presented as non-exposed / acid-exposed. B Two outliers were excluded for the differential expression and pathway enrichment analysis (BBE-017 and BBE-079). See Supplementary Table S12 for a phenotypical description of these patients. Fibro; fibroblasts.

Table S13. Quality report of RNA sequencing data from esophageal biopsy specimens.

Patient	Read count	Mapped to genes (%)

BBO-021	89,974,680	97.17
BBO-027	80,585,282	97.48
BBO-038	80,968,668	96.93
BBO-053	81,423,510	97.22
BBO-058	80,364,912	97.68
BBO-060	80,402,720	97.68
BBO-061	80,505,706	96.79
BBO-063	79,694,064	97.46
BBO-074	80,475,762	97.70
BBO-080	81,389,838	94.77
BBO-065	80,280,398	97.89
BBO-066	81,411,852	98.35
BBO-070	80,666,032	97.85
BBO-072	80,447,282	98.00
BBO-077	95,542,940	97.57
BBO-086	82,468,060	97.09
BBO-087	101,875,962	97.40
BBO-090	80,153,816	97.66
BBO-092	79,295,354	97.30
BBO-094	114,411,054	98.01
BBE-001	81,928,370	97.76
BBE-017	87,733,270	96.65
BBE-021	104,996,190	97.70
BBE-023	88,391,266	97.54
BBE-028	80,379,380	97.38
BBE-043	123,662,676	97.63
BBE-053	81,052,774	98.13
BBE-079	96,697,812	97.23
BBE-080	89,275,284	97.50
BBE-098	79,081,692	97.38
BBO-007	79,711,864	97.53
BBO-038	91,284,342	97.27
BBO-053	99,939,582	97.49
BBO-058	80,855,640	97.90
BBO-060	80,017,504	97.40
BBO-063	91,121,504	97.70
BBO-074	79,902,188	97.61
BBO-080	79,890,396	98.28
BBO-065	122,715,740	97.88
BBO-066	80,617,726	97.72
BBO-070	98,784,886	97.65
BBO-072	81,879,960	98.12
BBO-077	81,009,706	98.08
BBO-086	165,874,334	98.09
BBO-087	81,516,230	97.80
BBO-090	105,864,940	98.14
BBO-094	109,429,738	98.18
BBE-017	81,199,760	97.79
BBE-021	89,550,670	97.66
BBE-023	81,142,760	97.45
BBE-028	83,039,748	97.36
BBE-043	86,493,406	97.89
BBE-053	80,517,098	97.82

BBE-079	62,471,354	96.87
BBE-098	80,434,098	97.58

Table S14. Results of RNA isolation from fibroblast of the acid exposure experiments in terms of quantity and quality, plus a quality report of RNA sequencing data.

Sample	Concentration (ng/uL)	RIN	Read count	Mapped to genes (%)
Acid-exposed patient 1	30	7.3	128,303,494	98.93
Acid-exposed patient 2	70	9.2	103,308,260	98.99
Acid-exposed patient 3	30	8.3	87,959,820	98.93
Acid-exposed control 1	17	7.2	80,815,128	99.07
Acid-exposed control 2	66	8.3	80,630,850	99.06
Acid-exposed control 3	48	8.1	155,906,284	98.78
Non-exposed patient 1	239	9.3	101,489,322	94.55
Non-exposed patient 2	206	9.3	162,746,478	94.87
Non-exposed patient 3	141	9.8	121,773,766	94.59
Non-exposed control 1	31	9.0	131,980,322	95.82
Non-exposed control 2	223	9.3	134,581,046	95.67
Non-exposed control 3	173	9.0	124,560,450	95.10

RIN = RNA integrity number. See Supplementary Table S12 for a phenotypical description of these patients.

Table S15. Number of significantly differently expressed genes when comparing the different subgroups.

Comparison	Significant Genes (n)	Alternative Settings	Overlapping Disease and Developmental Genes [A]
I vs. II	3191		*BMP4, CDX1, CDX2, CFTR, EGFR, FOXF1, GATA6, HOXA13, MYC, PLCE1, SOX2*
I vs. III	20 [B]		-
I vs. IV	911		*BMP4, FOXF1, GATA6*
I vs. V	80 [B]		-
I vs. VI	5617		*BMP4, CCND1, CDX1, CDX2, CFTR, EGFR, FOXF1, GATA6, HOXA13, MYC, PLCE1, SOX2*
II vs. III	4446		*ABCC5, BMP4, CCND1, CDX1, CDX2, CFTR, EGFR, FOXF1, GATA6, HOXA13, MYC, PLCE1, SOX2*
II vs. IV	631 [B]		*CDX1, CDX2, CFTR, HOXA13, SOX2*
II vs. V	3657		*BMP4, CDX1, CDX2, CFTR, FOXF1, GATA6, HOXA13, MYC, PLCE1*
II vs. VI	981	FC <-3 or >3 → n = 514 [B]	-
III vs. IV	1677		*ABCC5, BMP4, FOXF1, GATA6*
III vs. V	165		-
III vs. VI	6090		*ABCC5, BMP4, CCND1, CDX1, CDX2, CFTR, EGFR, FOXF1, GATA6, GSTP, HOXA13, MYC, PLCE1, SOX2*
IV vs. V	973	FC <-3 or >3 → n = 521 [B]	*BMP4, FOXF1, GATA6*
IV vs. VI	3654		*BMP4, CCND1, CDX1, CDX2, CFTR, EGFR, FOXF1, GSTP, HOXA13, MYC, PLCE1, SOX2*
V vs. VI	5599		*BMP4, CDX1, CDX2, CFTR, FOXF1, GATA6, GSTP, HOXA13, MYC, PLCE1, SOX2*

Settings: max group mean >2, fold change (FC) <-1.5 or >1.5, false discovery rate (FDR) *p*-value <0.05. [A] Associated polymorphisms, previously found with genome

wide association studies (Supplementary Material S2) [B] Uploaded to Ingenuity Pathway Analysis. Group A = patients with esophageal atresia (EA) and Barrett's esophagus (BE), group B = patients with EA without BE, group C = patients with BE without EA in history, I = squamous cell epithelium (SQ) samples from group A, II = gastroesophageal junction (GEJ) samples from group A, III = SQ samples from group B, IV = GEJ samples from group B, V = SQ samples from group C, VI = GEJ samples from group C.

Table S16. Canonical pathways, significantly enriched by differentially expressed genes, and corresponding diseases and bio functions, with a significantly increased or decreased activations.

	**n = 353**	**-log(*p*-value)**	**z-score**
**Canonical pathways**	SPINK1 Pancreatic Cancer Pathway *	15.7	0
	Intrinsic Prothrombin Activation Pathway	7.92	1.897
	Neuroprotective Role of THOP1 in Alzheimer's Disease	6.83	3.207
	Cholecystokinin/Gastrin-mediated Signaling	4.38	2.111
	LXR/RXR Activation *	4.31	-2.111
	Toll-like Receptor Signaling #	3.73	1.89
	p38 MAPK Signaling #	3.72	1.897
	Adrenomedullin signaling pathway	3.56	2.309
	Acute Phase Response Signaling #	3.39	0.632
	IL-6 Signaling #	2.89	1.667
	PPAR Signaling	2.81	-1.414
	Production of Nitric Oxide and Reactive Oxygen Species in Macrophages #	2.68	0.302
	Dendritic Cell Maturation #	2.27	2.333
	Retinoate Biosynthesis I #	2.27	2
	HMGB1 Signaling #	2.09	1.633
	HIPPO signaling *	2.05	-1
	Retinol Biosynthesis #	1.95	-1
	Aryl Hydrocarbon Receptor Signaling *	1.51	-1
	Signaling by Rho Family GTPases #	1.49	2.333
	NF-κB Signaling #	1.45	2.121
	Sphingosine-1-phosphate Signaling # *	1.44	2.236
	Osteoarthritis Pathway #	1.44	-0.378
	Nicotine Degradation II	1.33	2
**Diseases and bio functions**	Migration of cells	2,00E-10	4.741
	Cell movement	9,38E-10	4.737
	Migration of tumor cell lines	4,85E-07	4.421
	Cell movement of tumor cell lines	6,48E-08	3.832
	Organization of cytoskeleton	3,57E-04	3.733
	Organization of cytoplasm	2,07E-03	3.733
	Invasion of tumor cell lines	6,79E-06	3.658
	Cell movement of blood cells	1,42E-04	3.430
	Leukocyte migration	2,01E-04	3.344
	Cell movement of leukocytes	8,70E-05	3.331
	Chemotaxis	6,96E-05	3.209
	Chemotaxis of leukocytes	1,13E-04	3.108
	Cell movement of myeloid cells	8,49E-04	3.071
	Invasion of cells	2,53E-08	3.057
	Homing of cells	9,80E-05	3.054
	Formation of skin	5,53E-40	3.042
	Chemotaxis of myeloid cells	1,92E-04	2.939
	Inflammatory response	4,46E-07	2.803
	Chemotaxis of phagocytes	1,08E-04	2.690

Cell movement of breast cancer cell lines	2,56E-04	2.573
Formation of epidermis	5,06E-24	2.433
Neoplasia of tumor cell lines	2,69E-05	2.384
Cell movement of mononuclear leukocytes	1,69E-03	2.280
Differentiation of epithelial cells	3,23E-19	2.269
Cell movement of granulocytes	3,01E-05	2.263
Chemotaxis of granulocytes	3,52E-05	2.260
Chemotaxis of neutrophils	3,05E-04	2.254
Cell proliferation of carcinoma cell lines	2,31E-05	2.186
Advanced malignant tumor	9,25E-04	2.161
Neoplasia of cells	2,25E-04	2.144
Activation of phagocytes	2,94E-05	2.119
Cancer of cells	5,83E-04	2.114
Differentiation of skin	1,11E-24	2.064
Metabolism of eicosanoid	5,05E-04	2.026
Allergy	2,20E-13	2.019
Weight loss	7,35E-04	-2.030
Blister	1,71E-04	-2.219
Apoptosis of skin	3,39E-04	-2.595
Congenital anomaly of digit	6,28E-06	-2.949
Limb defect	1,70E-05	-3.110
Congenital anomaly of limb	2,57E-07	-3.110

Settings: p-value <0.05 (= -log(p-value) > 1.3), Z-score <-2 or >2 (only for diseases and bio functions). n = total number of canonical pathways significantly enriched by differentially expressed genes, N/A = not applicable, z-score could not be calculated. Group A = patients with esophageal atresia (EA) and Barrett's esophagus (BE), group C = patients with BE without EA in history, I = squamous cell epithelium (SQ) samples from group A, II = gastroesophageal junction (GEJ) samples from group A, III = SQ samples from group B, IV = GEJ samples from group B, V = SQ samples from group C, VI = GEJ samples from group C. * = involved in oncological processes, [#] = involved inflammatory processes.

Table S17. Canonical pathways, enriched by differentially expressed genes.

	**Canonical Pathways**	**-log(*p*-value)**	**z-score**
**Acid-exposed vs. non-exposed (all samples) (n = 578) [A]**	EIF2 Signaling	43.3	5.338
	Coronavirus Pathogenesis Pathway	19	-2.722
	Oxidative Phosphorylation	15.8	5.642
	Kinetochore Metaphase Signaling Pathway	14.8	-3.452
	Sirtuin Signaling Pathway	9.8	-3
	Cell Cycle: G2/M DNA Damage Checkpoint Regulation	7.99	2.982
	Unfolded protein response	6.84	2.183
	NRF2-mediated Oxidative Stress Response	6.51	2.683
	IL-6 Signaling	5.02	2.117
	ILK Signaling	3.41	3.413
	RAN Signaling	3.17	-2.646
	Death Receptor Signaling	2.89	-2.065
	Hypoxia Signaling in the Cardiovascular System	2.74	2.646
	BAG2 Signaling Pathway	264	2.111
	PPAR Signaling	2.57	-2.524
	PCP pathway	2.33	2.309
	Cell Cycle Control of Chromosomal Replication	2.15	-3.464
	IL-17 Signaling	2.07	3.157
	Role of PKR in Interferon Induction and Antiviral Response	1.98	-2.236
	Inhibition of ARE-Mediated mRNA Degradation Pathway	1.83	3.130

	MIF Regulation of Innate Immunity	1.74	2.333
	Angiopoietin Signaling	1.53	-2.121
	Regulation Of The Epithelial Mesenchymal Transition In Development Pathway	1.48	2.496
**Patients vs. controls (acid-exposed) (n = 258)** [B]	Dendritic Cell Maturation	4.6	-0.707
	Type I Diabetes Mellitus Signaling	4.09	N/A
	T Helper Cell Differentiation	3.97	N/A
	Role of Macrophages, Fibroblasts and Endothelial Cells in Rheumatoid Arthritis	3.71	N/A
	Hepatic Fibrosis / Hepatic Stellate Cell Activation	3.7	N/A
	Graft-versus-Host Disease Signaling	3.6	N/A
	Role of Osteoblasts, Osteoclasts and Chondrocytes in Rheumatoid Arthritis	3.28	N/A
	PD-1, PD-L1 cancer immunotherapy pathway	3.22	1.342
	Axonal Guidance Signaling	2.88	N/A
	Coagulation System	2.84	N/A
**Patients vs. controls (non-exposed) (n = 314)** [B]	Osteoarthritis Pathway	6.98	-1.265
	Hepatic Fibrosis / Hepatic Stellate Cell Activation	5.99	N/A
	Tumor Microenvironment Pathway	5.34	0
	Axonal Guidance Signaling	4.96	N/A
	HOTAIR Regulatory Pathway	4.82	-1
	Role of Osteoblasts, Osteoclasts and Chondrocytes in Rheumatoid Arthritis	4.53	N/A
	Colorectal Cancer Metastasis Signaling	3.99	-0.333
	Role of Macrophages, Fibroblasts and Endothelial Cells in Rheumatoid Arthritis	3.87	N/A
	Caveolar-mediated Endocytosis Signaling	3.29	N/A
	HIF1α Signaling	3.26	-0.707

[A] Settings: *p*-value <0.05 (= -log(*p*-value) > 1.3), z-score <-2 or >2. [B] Settings: *p*-value <0.05 (= -log(*p*-value) > 1.3), top 10 pathways presented. n = total number of canonical pathways significantly enriched by differentially expressed genes. N/A = not applicable, z-score could not be calculated.

Table S18. Canonical pathways, significantly enriched by differentially expressed genes.

Canonical Pathways (n = 173)	-log(*p*-value)	z-score
Communication between Innate and Adaptive Immune Cells #	4.85	N/A
Graft-versus-Host Disease Signaling #	4.77	N/A
Dendritic Cell Maturation #	4.56	0.816
LXR/RXR Activation *	4.36	-2.236
Role of Hypercytokinemia/hyperchemokinemia in the Pathogenesis of Influenza #	3.77	0
Altered T Cell and B Cell Signaling in Rheumatoid Arthritis #	3.70	N/A
Hepatic Fibrosis Signaling Pathway	3.69	1.89
Granulocyte Adhesion and Diapedesis #	3.63	N/A
PPAR Signaling	3.44	-2
Airway Pathology in Chronic Obstructive Pulmonary Disease	3.25	N/A
IL-6 Signaling #	3.14	2
Atherosclerosis Signaling	3.13	N/A
LPS/IL-1 Mediated Inhibition of RXR Function #	3.11	N/A
IL-10 Signaling #	2.83	N/A
T Helper Cell Differentiation #	2.78	N/A
TR/RXR Activation #	2.60	N/A
Hepatic Cholestasis	2.52	N/A
Hepatic Fibrosis / Hepatic Stellate Cell Activation	2.52	N/A
PD-1, PD-L1 cancer immunotherapy pathway *	2.32	N/A

Role of Osteoblasts, Osteoclasts and Chondrocytes in Rheumatoid Arthritis	2.28	N/A
Type I Diabetes Mellitus Signaling	2.26	N/A
Coagulation System	2.25	N/A
B Cell Development #	2.23	N/A
p38 MAPK Signaling #	2.19	N/A
Antigen Presentation Pathway #	2.16	N/A
FXR/RXR Activation # *	2.11	N/A
Intrinsic Prothrombin Activation Pathway	2.10	N/A
Th2 Pathway #	2.02	N/A
Autoimmune Thyroid Disease Signaling #	1.97	N/A
Role of Cytokines in Mediating Communication between Immune Cells #	1.89	N/A
Neuroinflammation Signaling Pathway #	1.80	-2
HMGB1 Signaling #	1.80	N/A
Th1 and Th2 Activation Pathway #	1.76	N/A
Role of Macrophages, Fibroblasts and Endothelial Cells in Rheumatoid Arthritis	1.74	N/A
Calcium-induced T Lymphocyte Apoptosis # *	1.72	N/A
NF-κB Signaling #	1.70	N/A
Acute Phase Response Signaling #	1.70	N/A
Role of NFAT in Regulation of the Immune Response #	1.69	N/A
Axonal Guidance Signaling	1.68	N/A
Regulation Of The Epithelial Mesenchymal Transition By Growth Factors Pathway *	1.65	N/A
Clathrin-mediated Endocytosis Signaling *	1.62	N/A
Agranulocyte Adhesion and Diapedesis #	1.62	N/A
TREM1 Signaling #	1.62	N/A
Toll-like Receptor Signaling #	1.61	N/A
BEX2 Signaling Pathway *	1.58	N/A
Pathogenesis of Multiple Sclerosis	1.55	N/A
FGF Signaling *	1.53	N/A
IL-4 Signaling #	1.52	N/A
Allograft Rejection Signaling #	1.51	N/A
Crosstalk between Dendritic Cells and Natural Killer Cells #	1.48	N/A
Osteoarthritis Pathway #	1.47	N/A
OX40 Signaling Pathway #	1.47	N/A
Bladder Cancer Signaling *	1.41	N/A
Bile Acid Biosynthesis, Neutral Pathway	1.39	N/A
iCOS-iCOSL Signaling in T Helper Cells #	1.31	N/A

Settings: *p*-value <0.05 (= -log(*p*-value) > 1.3), n = total number of canonical pathways significantly enriched by differenially expressed genes. N/A = not applicable, z-score could not be calculated. * = involved in oncological processes, # = involved inflammatory processes.

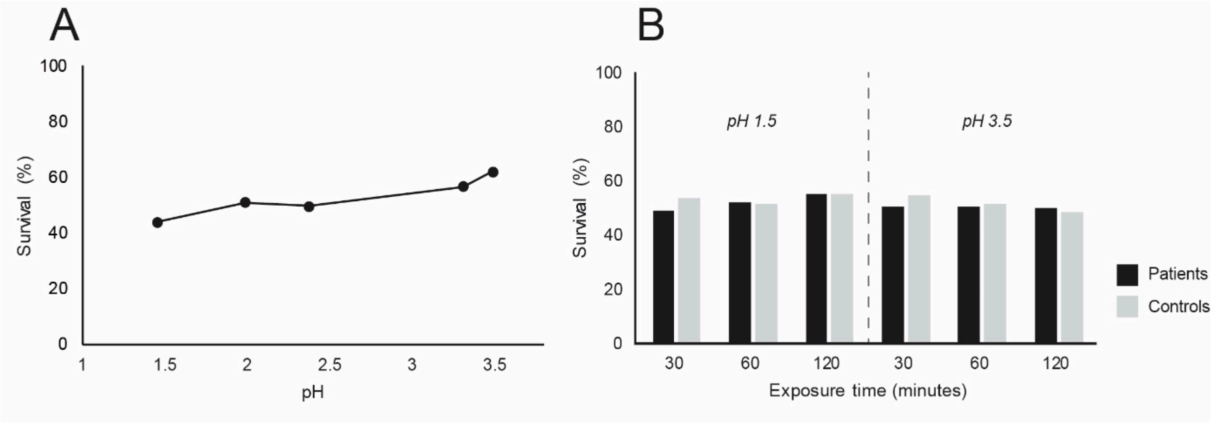

Figure S1. Overview of survival rates of fibroblast cells after exposure to pH adjusted medium.

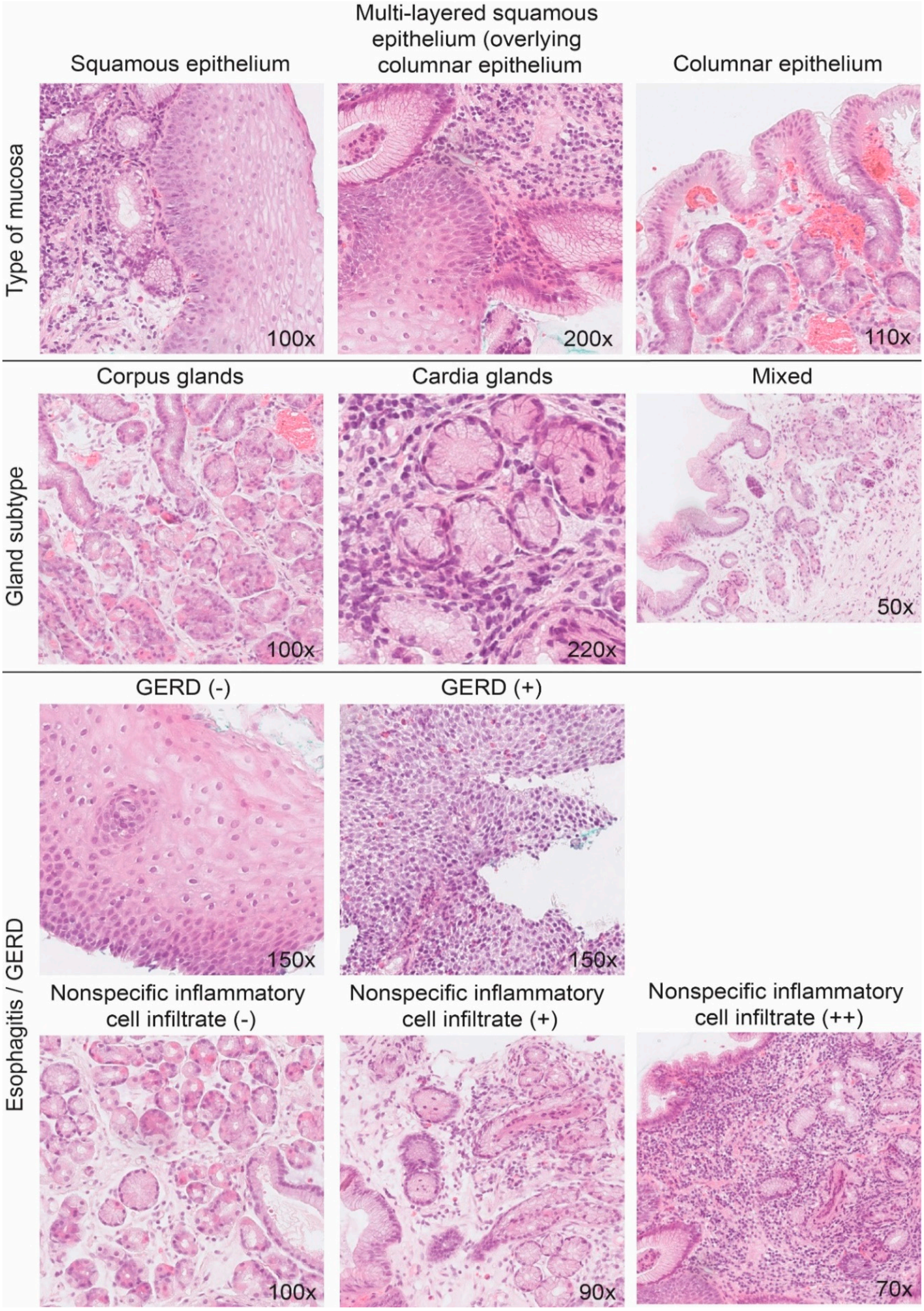

A

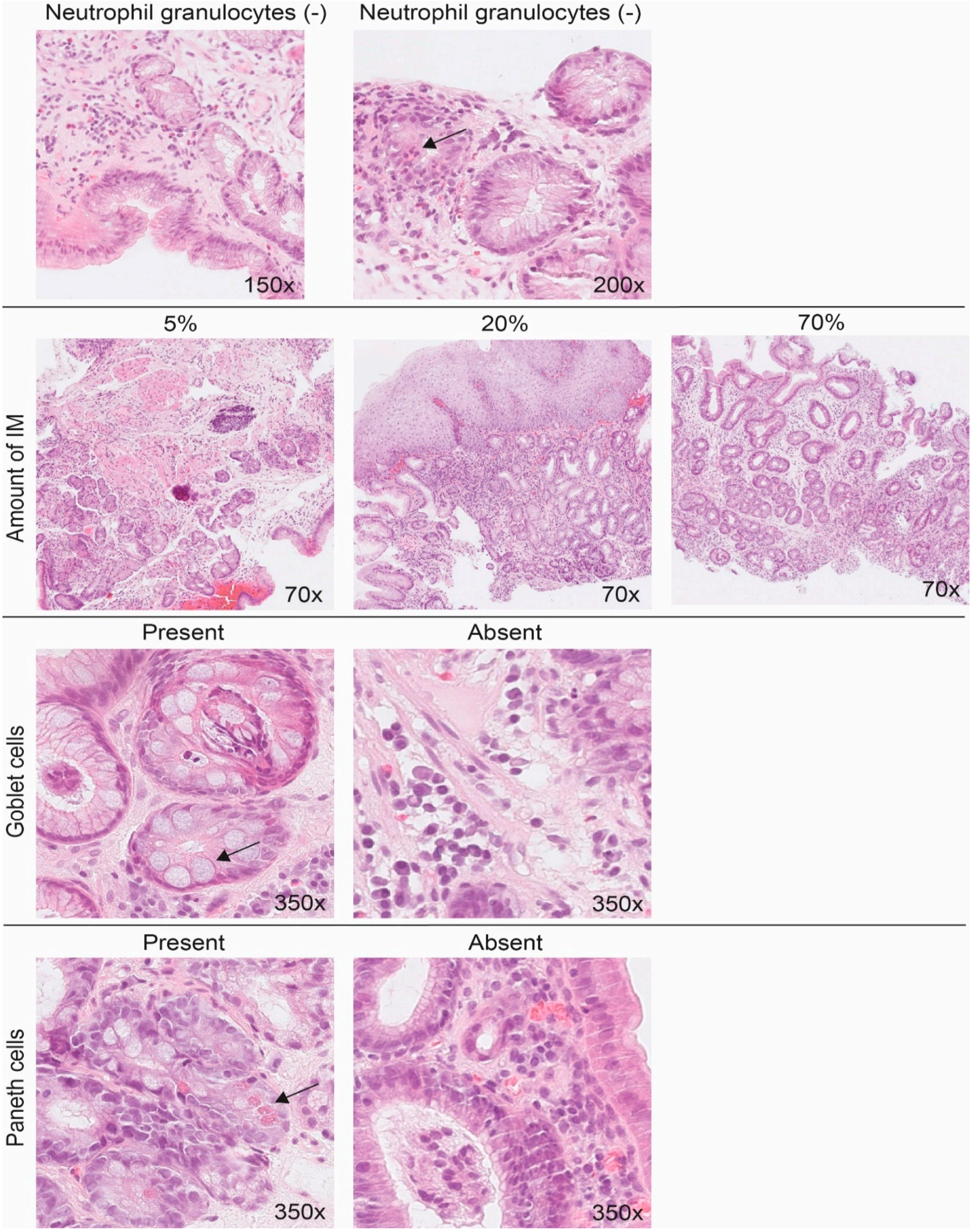
Neutrophil granulocytes (-)
Neutrophil granulocytes (-)
150x
200x
Amount of IM
5%
20%
70%
70x
70x
70x
Goblet cells
Present
Absent
350x
350x
Paneth cells
Present
Absent
350x
350x

B

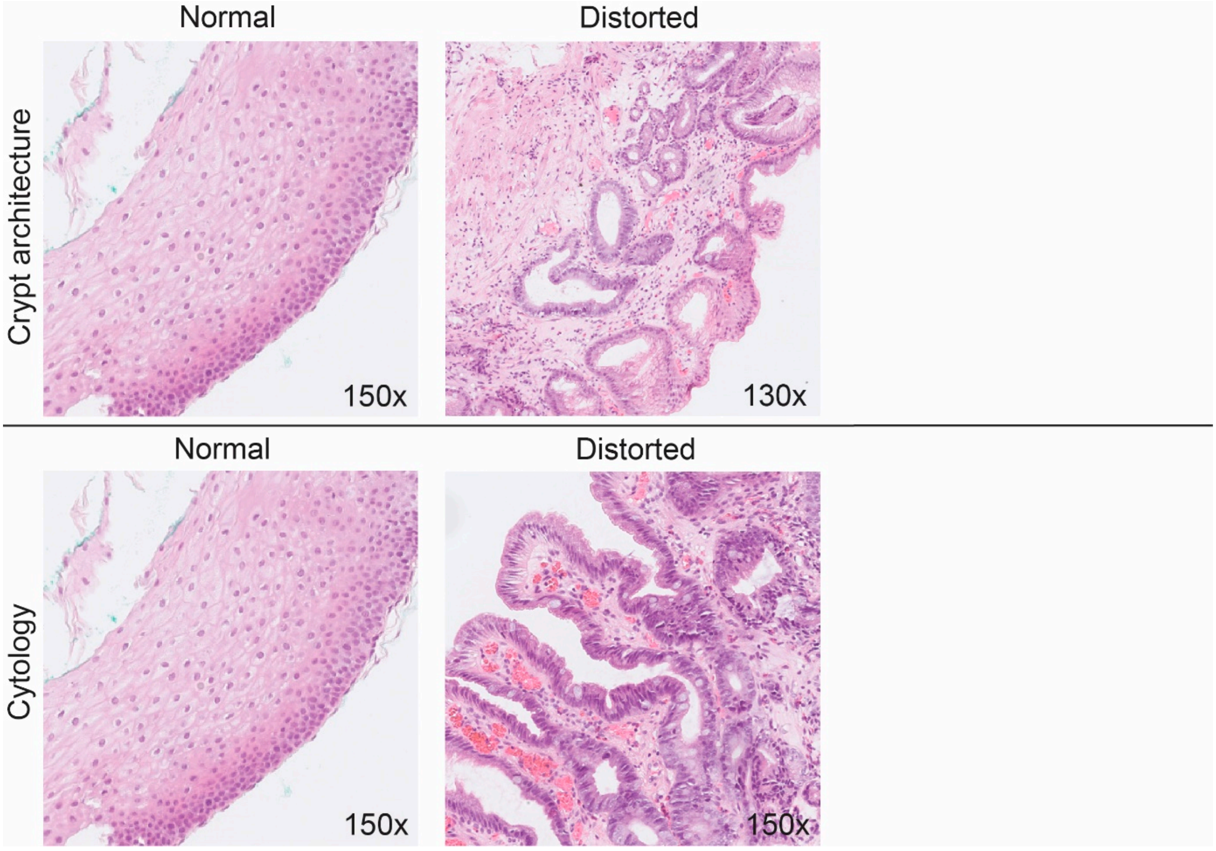

C

Figure S2. part A. Examples of the review-based checklist [6], used for the histopathological assessments of pathology slides of esophageal biopsy specimens, with the magnification of each image. part B. Examples of the review-based checklist [6], used for the histopathological assessments of pathology slides of esophageal biopsy specimens, with the magnification of each image. part C. Examples of the review-based checklist [6], used for the histopathological assessments of pathology slides of esophageal biopsy specimens, with the magnification of each image.

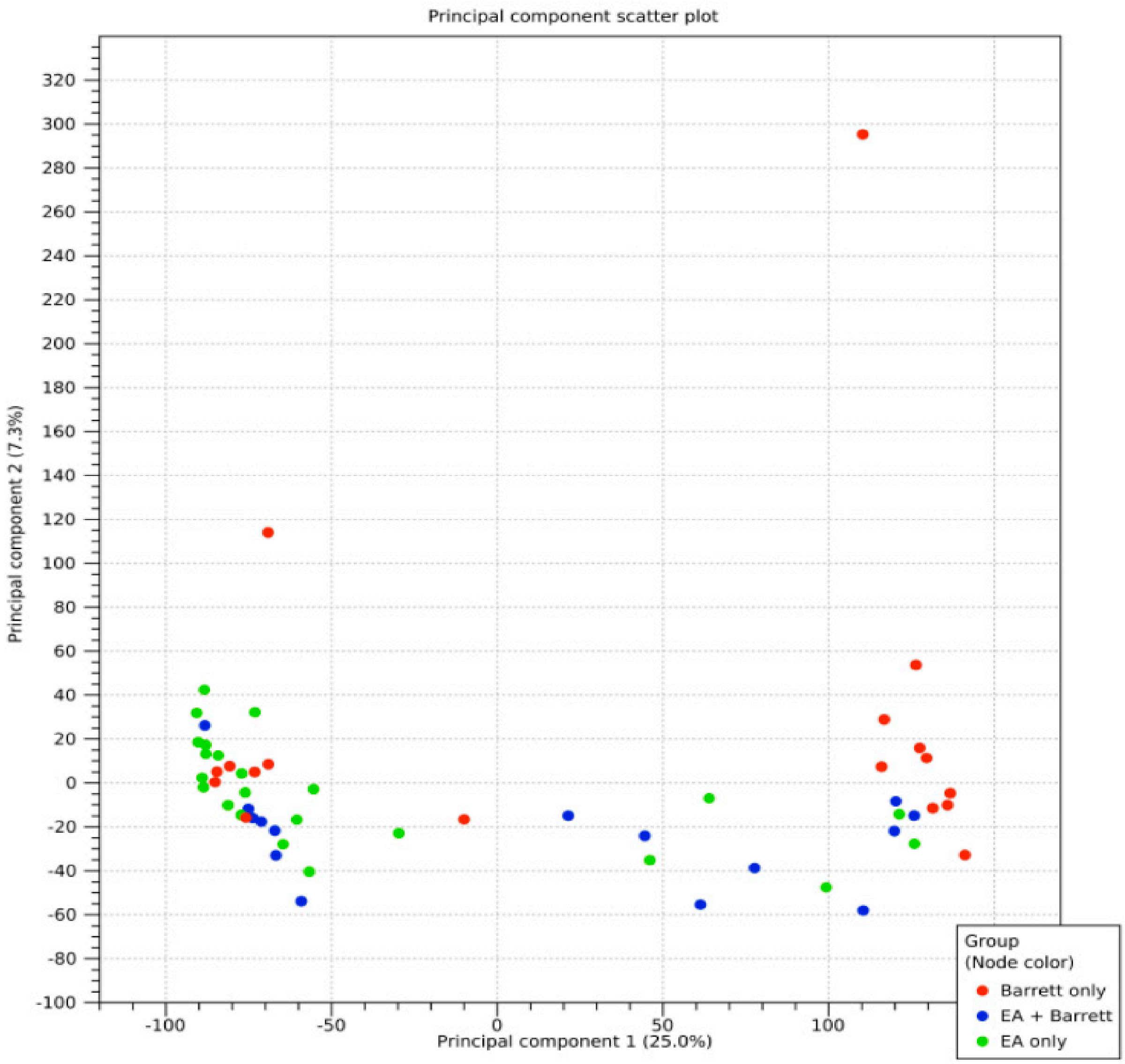

Figure S3. Two-dimensional scatter plot of principal component analysis (PCA), clustered for group. The percentages represent the proportion of variant explained by that specific principal component. Two outliers (BBE-017 and BBE-079) were excluded for further analysis. EA = esophageal atresia.

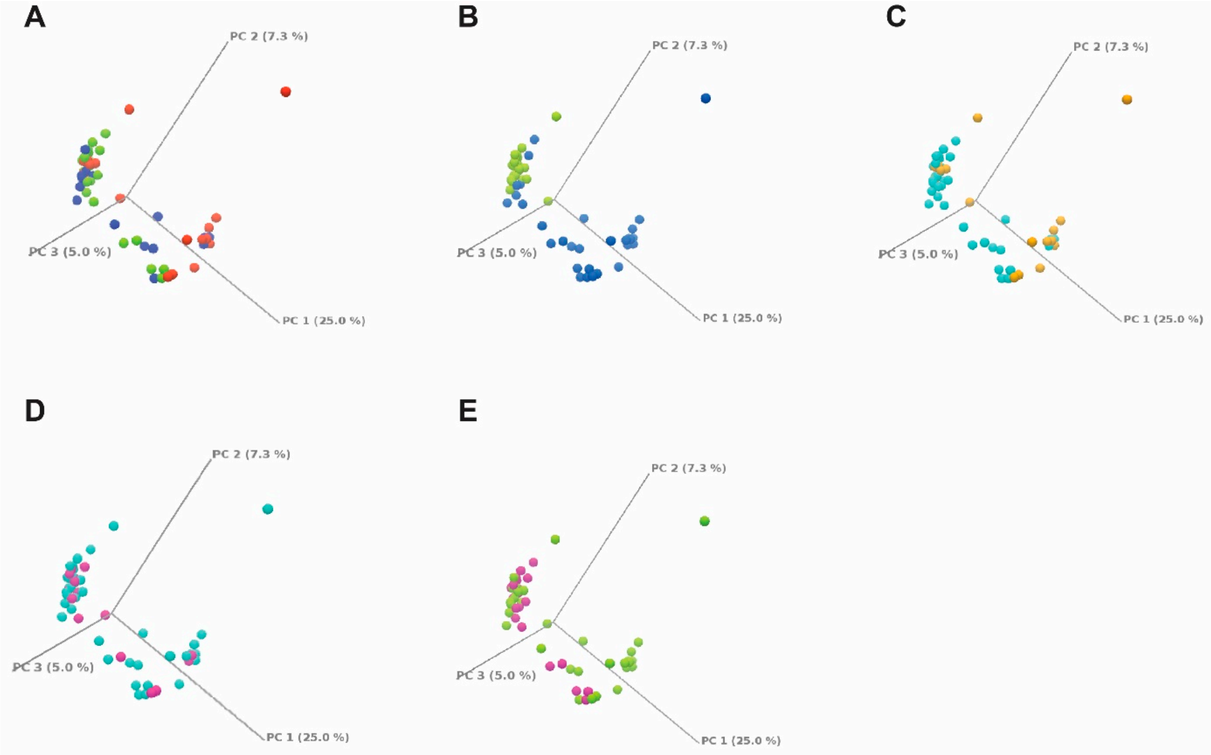

Figure S4. Tree-dimensional scatter plot of principal component analysis (PCA), clustered for A = group (esophageal atresia (EA) with Barrett's esophagus (BE); EA only; and BE only), B = origin of biopsy, C = EA in history, D = sex, and E = presence of BE.

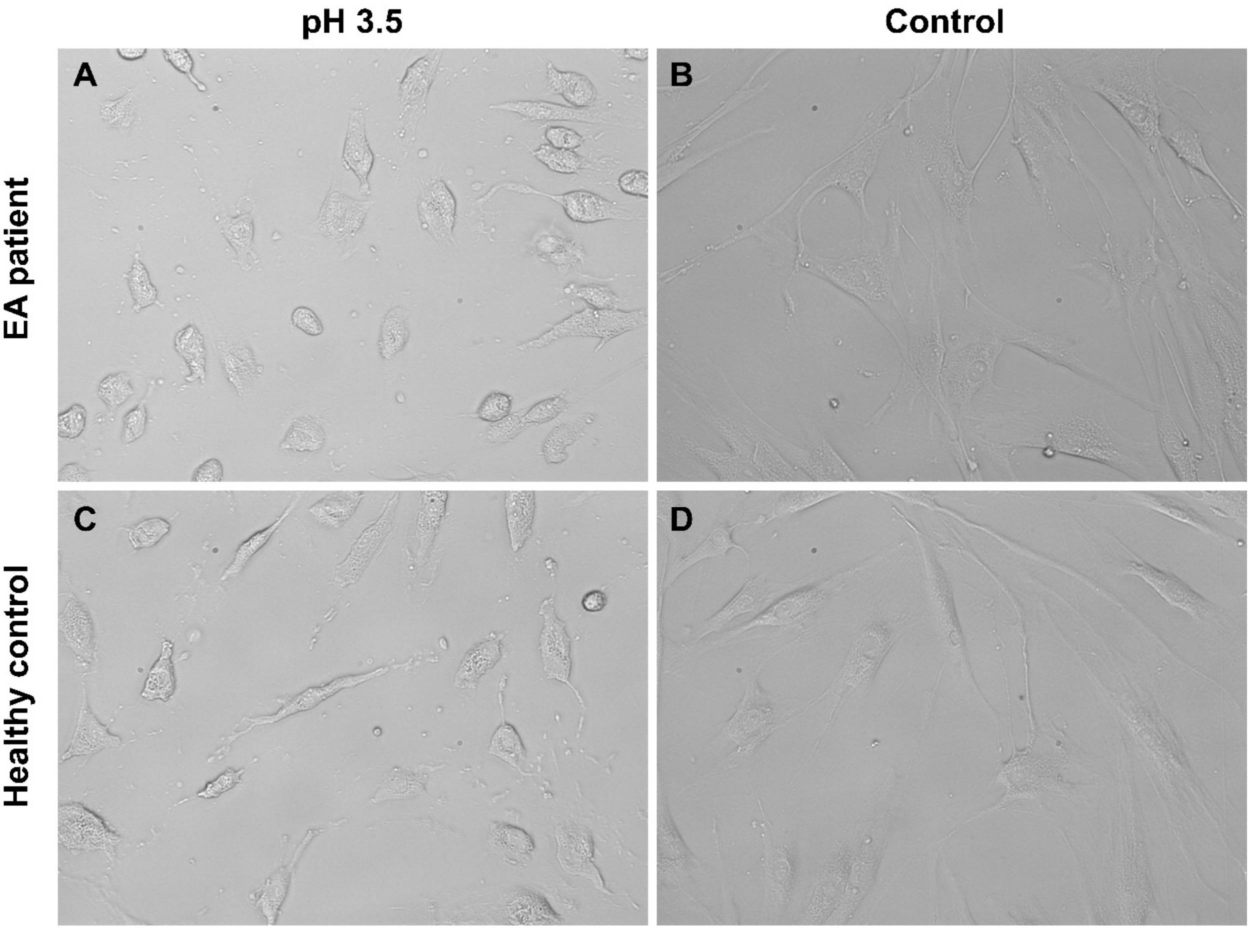

Figure S5. Morphology (20x magnification) of fibroblast cells after 30 minutes exposure to acid Dulbecco's Modified Eagle's Medium with pH 3.5 and control medium, of a patient with esophageal atresia (EA, A and B) and a healthy control (C and D).

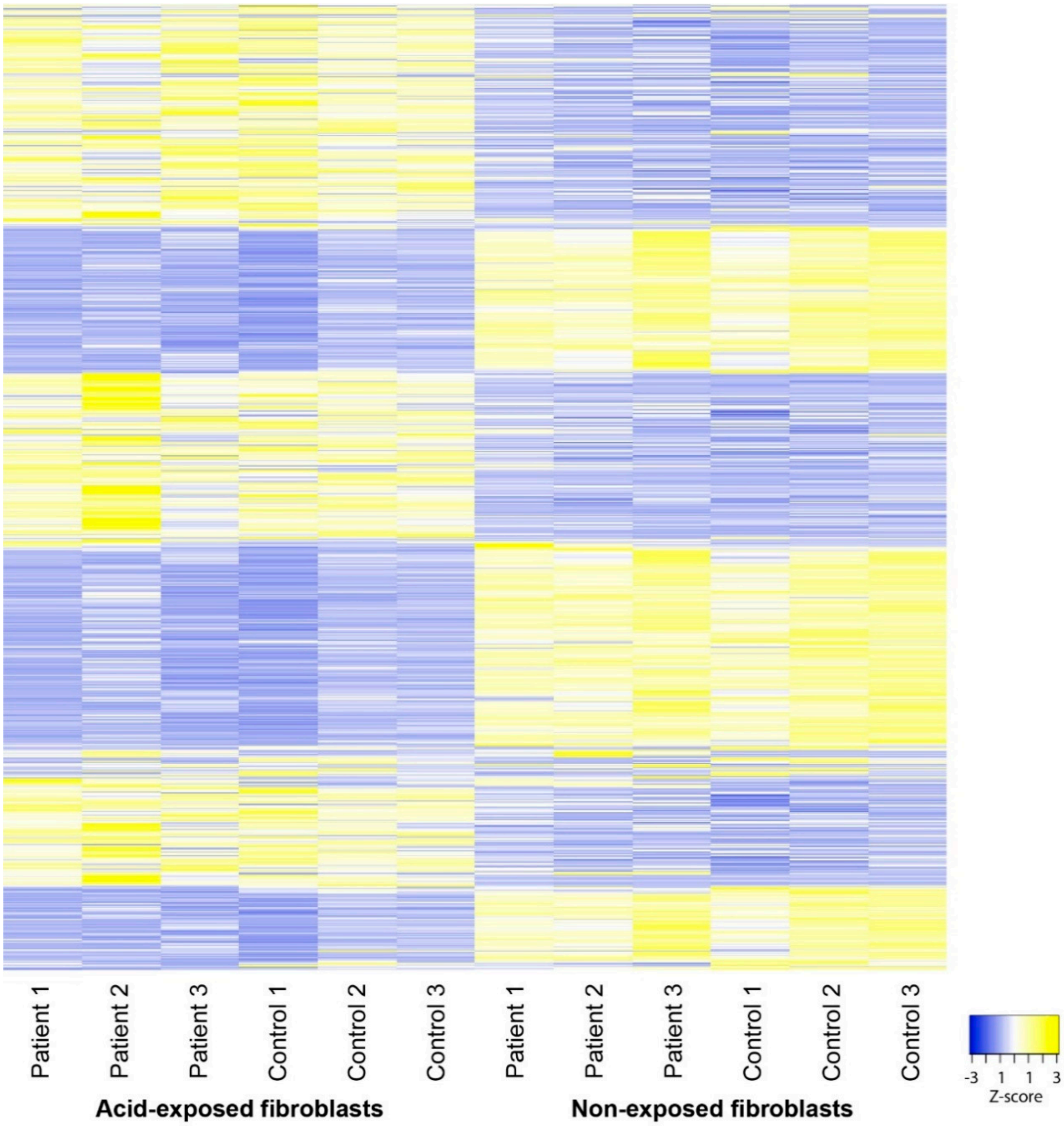

Figure S6. Heat map of mean transcript per million (TPM) for fibroblast cells after the in vitro experiment, for a selected gene panel (n = 2344). We evaluated all genes (n = 2344) of the enriched pathways between GEJ samples of EA/BE patients and GEJ samples of BE only patients. For these 2344 genes, there was a clear difference between upregulated and downregulated genes in fibroblasts after acid exposure, in both EA patients and healthy controls. Gene panel was extracted from the significantly enriched pathways between gastroesophageal junction (GEJ) samples of group A (esophageal atresia (EA) with Barrett's esophagus (BE)) and GEJ samples of group C (BE only), see Supplementary Table S14. Settings: maximum group mean >2, fold change <-1.5 or >1.5, false discovery rate *p*-value <0.05, hierarchical clustering by average linkage, distance between rows and columns by Eucledian method.

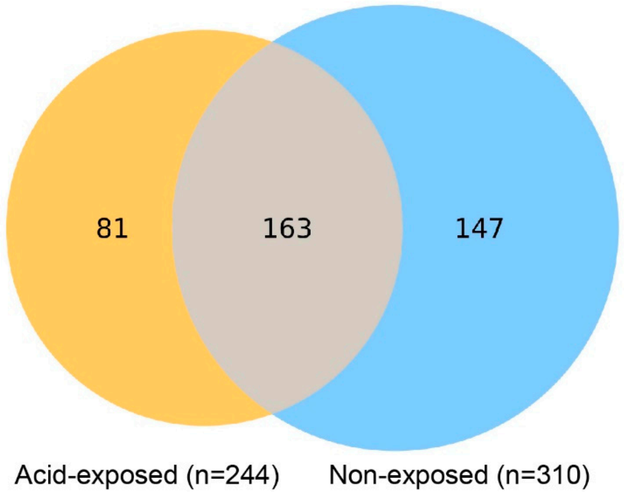

Figure S7. Number of differentially expressed genes between patients with EA and healthy controls after acid-exposure (yellow, n = 244), and number of differentially expressed genes between patients and controls without exposure (blue, n = 310). This leaves 81 genes that were differentially expressed between patients and controls after acid exposure but NOT without exposure.

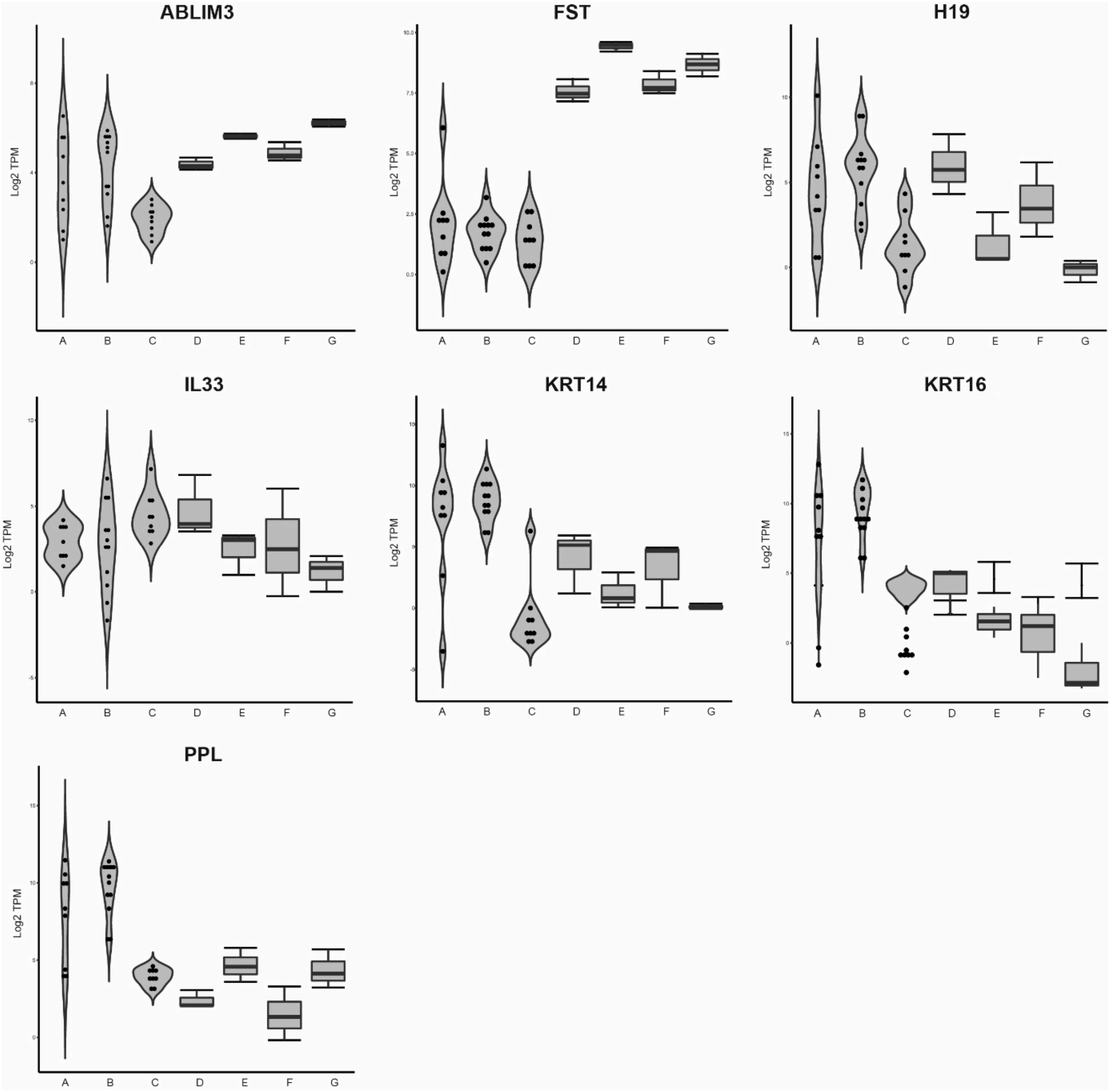

Figure S8. Violin plots of gastroesophageal (GEJ) samples (left) and box plots of acid-exposed fibroblasts (right). Of the overlapping enriched pathways of GEJ samples of patients with esophageal atresia (EA) who have developed Barrett's esophagus (BE) compared to BE only patients and acid-exposed fibroblasts of patients compared to controls, seven genes were differentially expressed in both GEJ samples and in fibroblasts. A = EA/BE patients, B = EA only patients, C = BE only, D = EA patients (acid-exposed), E = controls (acid-exposed), F = EA patients (non = exposed), G = Controls (non-exposed). TPM = transcripts per million.

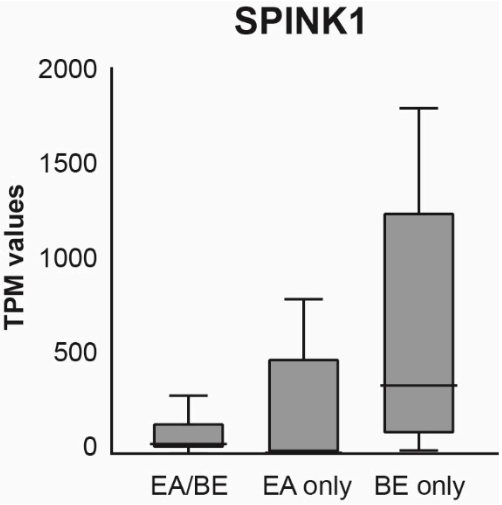

Figure S9. SPINK1 is a potential biomarker for Barrett's esophagus. SPINK1 Pancreatic Cancer Pathway was downregulated in EA/BE patients compared with BE only patients (Z-score = -3, $p < 0.0001$). SPINK1 is an enzyme secreted by pancreatic acinar cells, and it is a clinical indicator of malignant disease [72]. The pancreas is derived from the distal foregut, and some of the involved transcription factors during overlap with those of esophageal development [73]. *SPINK1* is expressed in the liver, pancreas and the gastrointestinal tract, with increased expression in gastrointestinal tumors [72]. Although it does not seem to be expressed in unaffected esophagus [74], *SPINK1* was expressed in the majority of the patients with highest TPM levels in BE only patients (Of note: *SPINK1* is somewhat expressed in the stomach [74]. Biopsies of EA only patients were taken from the gastroesophageal junction, which means that biopsies could contain some stomach tissue as well.

SM1: Sample extraction protocol and storage

All materials used in this study were retrieved from the Biobank Esophageal Atresia (MEC-2015-645) and the Biobank Barrett (MEC-2010-094), in which samples have been stored after written informed consent. During surveillance endoscopies, 2x2 mucosal biopsies were taken from two esophageal sites, one for histological evaluation and one for RNA extraction. The first from the unaffected esophageal squamous cell epithelium (SQ). In patients with EA this biopsy was taken above the original anastomosis. The second set of biopsies was taken from the GEJ or, if present, from Barrett's mucosa (Figure 3 in main manuscript). All biopsies either have been snap frozen in liquid nitrogen directly and stored at -80°C, or have been transferred in a RNAlater™ Stabilization Solution (Thermo Fisher Scientific, Waltham, USA) overnight at 4°C, after which the RNAlater was removed and the biopsies were stored at -80°C. EDTA blood samples have been collected and stored at -20°C before extraction of genomic DNA.

SM2: RNA and DNA isolation

DNA was extracted from peripheral blood and fibroblasts using the DNA Mini Kit (Qiagen, Venlo, the Netherlands). DNA quality and quantity was determined with the Thermo Scientific Nano Drop 2000 (ThermoFisher Scientific Inc., Waltham, USA) and Quant-iT™ PicoGreen ® dsDNA kit (Invitrogen, Carlsbad, CA, USA). Total RNA was isolated from the biopsies and fibroblasts using the AllPrep DNA/RNA Mini Kit (Qiagen, Venlo, the Netherlands) or the RNeasy Mini Kit (Qiagen, Venlo, the Netherlands) or the E.Z.N.A.® Total RNA Kit I (Omega Bio-tek Inc., Norcross, Georgia, USA), and stored at -80°C. The quantity and quality of the RNA was determined with the Lab-on-Chip RNA 6000 Nano (Agilent Technologies, Santa Clara, USA). Samples with an RNA integrity number (RIN) >6 were prepared and processed for RNA sequencing.

SM3: SNP genotyping

A total of 200 ng dsDNA was used for single nucleotide polymorphism (SNP) array genotyping analysis using the Infinium Global Screening Array v1.0 or v3.0 (Illumina, Inc. San Diego, USA), according to the manufacturer's standard protocol. Output was generated using Illumina Genome studio v2.0 (Illumina, San Diego, CA, USA). Using SNP-array, we also evaluated DNA copy number variations (CNV) profiles of the biopsies of EA/BE patients and BE patients, if sufficient amounts and quality of DNA was present. We determined genomic stability (the presence or absence of large de novo gains or losses) by inspecting these visually in Biodiscovery Nexus CN10.0 (Biodiscovery Inc., Hawthorne, CA, USA) and comparing them to their germline counterpart. Predisposition loci (and corresponding lead or proxy SNPs) associated with BE, EAC and/or ESCC were derived from the literature (see Supplementary Material S2) [55–69]. We used SNP array genotyping data from patients of group A (EA/BE, n = 19), patients of group B (EA only, n = 44) and patients of group C (BE only, n = 10) to see if previously BE associated SNPs and/or haplotypes were more prevalent in patients with EA and BE. If the associated SNP was not present on the genotyping platform, a proxy was selected using the LD proxy and LD pair Tool (https://ldlink.nci.nih.gov,accessed on 2021/10/15), with a cut-off level of

D′ > 0.9 and R′ > 0.6. We compared these genotypes to unaffected controls (n = 730), sequenced on the same platform as our patients, with a chi-square test and calculated odds ratios (ORs). Admixture was used to infer ancestry [75]. We used the allele counts and published ORs of the associated SNPs to calculate a polygenic risk score (PGRS) using an additive model: $PGRS = \sum Ln\ (OR\ risk\ allele) * allele\ count$ [76]. If multiple studies published an OR for a SNP, the OR from the study with the largest sample size was included in the PGRS. In a second calculation, we used the ORs of the associated SNPs as calculated from our study population. A Kruskal-Wallis test and Mann-Whitney tests were used to compare the PGRS between the different groups. All statistical analyses were performed in SPSS V.25.0 (IBM, Chicago, Illinois, USA), with a significance level of $p < 0.05$.

SM4: RNA sequencing

First, strand cDNA libraries were made with the strand-specific NEBNext Ultra II Directional RNA Library Prep Kit protocol and polyA mRNA workflow (NEB #E7760S/L) on an Illumina NovaSeq6000 (Illumina, San Diego, USA). Quality control, read trimming, read alignment, transcript quantification and differential expression analysis were performed using CLC Genomics Workbench version 20 (Qiagen, Venlo, The Netherlands). Reads were aligned to the human reference genome (hg19) according to the following settings: mismatch cost 2, insertion/deletion cost 3, length fraction 0.8, similarity fraction 0.8, alignment to gene regions only. Paired reads were counted as one. Trimmed mean per million (TMM) values was used to normalize for sequencing depth across samples. For each gene, counts per million (CPM) and transcripts per million (TPM) were calculated. Read counts were normalized for transcript length and total number of mapped reads (RPKM). Principal component analysis (PCA) was performed to explore cluster separation and identify outlying samples [77]. Counts for each individual gene were transformed to a smaller set of orthogonal principal components, in which the first component specifies the direction with the largest variability in the data. Two-dimensional and three-dimensional plots are produced. For each gene, log CPM values and a Z-score were calculated using all samples. Our ethics committee does not allow sharing of individual patient or control genotype information in the public domain, including sequencing reads.

SM5: Acid exposure experiments

To study the effect of GER on RNA level, we simulated a reflux episode in an in vitro experiment (Figure 3 in the main manuscript). The pH level of the stomach normally varies between 1.0 and 3.5 [78]. We evaluated the survival of fibroblasts in medium with a pH level between pH 1.5 and 3.5. All experiments were performed in duplo. Survival rate differences were determined using a paired t-test or a one-way analysis of variance (ANOVA). Next, comparison of an exposure time of 30, 60 and 120 minutes in both patient and control cell lines using these pH levels did not show a significant difference (Figure S1 and Table S7). Since a reflux episode is usually several minutes, we continued with an exposure time of 30 minutes.

For the final experiment, we exposed human fibroblasts from three patients with EA and three healthy controls for 30 minutes to medium with a pH level of 3.5 or to normal medium (control). Human fibroblasts were cultured in DMEM (Dulbecco's Modified Eagle's Medium; Invitrogen, Carlsbad, CA, USA), supplemented with 10% fetal calf serum (FCS) and 1% penicillin/streptomycin at 37 °C under a humidified 5% CO_2 atmosphere. Hydrochloric acid was added to culture medium until the desired pH level was reached. We selected an exposure time of 30 minutes to pH 3.5 adjusted medium. Subsequently, cells were washed with phosphate buffered saline (PBS) and given standard medium. After 24 hours, survival was measured with the TC20™ Automated Cell Counter (Bio-Rad Laboratories B.V., Veenendaal, The Netherlands). Cell morphology was evaluated with the Olympus IX70-S8F Inverted Fluorescence Microscope (Olympus Corporation, Tokyo, Japan).

References

55. Palles, C.; Chegwidden, L.; Li, X.; Findlay, J.M.; Farnham, G.; Castro Giner, F.; Peppelenbosch, M.P.; Kovac, M.; Adams, C.L.; Prenen, H., et al. Polymorphisms near tbx5 and gdf7 are associated with increased risk for barrett's esophagus. *Gastroenterology* **2015**, *148*, 367–378.
56. Levine, D.M.; Ek, W.E.; Zhang, R.; Liu, X.; Onstad, L.; Sather, C.; Lao-Sirieix, P.; Gammon, M.D.; Corley, D.A.; Shaheen, N.J., et al. A genome-wide association study identifies new susceptibility loci for esophageal adenocarcinoma and barrett's esophagus. *Nat Genet* **2013**, *45*, 1487–1493.
57. Casson, A.G.; Zheng, Z.; Evans, S.C.; Geldenhuys, L.; van Zanten, S.V.; Veugelers, P.J.; Porter, G.A.; Guernsey, D.L. Cyclin d1 polymorphism (g870a) and risk for esophageal adenocarcinoma. *Cancer* **2005**, *104*, 730–739.
58. Ren, D.; Zheng, G.; Bream, S.; Tevebaugh, W.; Shaheen, N.J.; Chen, X. Single nucleotide polymorphisms of caudal type homeobox 1 and 2 are associated with barrett's esophagus. *Dig Dis Sci* **2014**, *59*, 57–63.
59. Su, Z.; Gay, L.J.; Strange, A.; Palles, C.; Band, G.; Whiteman, D.C.; Lescai, F.; Langford, C.; Nanji, M.; Edkins, S., et al. Common variants at the mhc locus and at chromosome 16q24.1 predispose to barrett's esophagus. *Nat Genet* **2012**, *44*, 1131–1136.
60. Becker, J.; May, A.; Gerges, C.; Anders, M.; Veits, L.; Weise, K.; Czamara, D.; Lyros, O.; Manner, H.; Terheggen, G., et al. Supportive evidence for foxp1, barx1, and foxf1 as genetic risk loci for the development of esophageal adenocarcinoma. *Cancer Med* **2015**, *4*, 1700–1704.
61. Kala, Z.; Dolina, J.; Marek, F.; Izakovicova Holla, L. Polymorphisms of glutathione s-transferase m1, t1 and p1 in patients with reflux esophagitis and barrett's esophagus. *Journal of human genetics* **2007**, *52*, 527–534.
62. Bull, L.M.; White, D.L.; Bray, M.; Nurgalieva, Z.; El-Serag, H.B. Phase i and ii enzyme polymorphisms as risk factors for barrett's esophagus and esophageal adenocarcinoma: A systematic review and meta-analysis. *Dis Esophagus* **2009**, *22*, 571–587.
63. McElholm, A.R.; McKnight, A.J.; Patterson, C.C.; Johnston, B.T.; Hardie, L.J.; Murray, L.J.; Finbar, G. A population-based study of igf axis polymorphisms and the esophageal inflammation, metaplasia, adenocarcinoma sequence. *Gastroenterology* **2010**, *139*, 204–212 e203.
64. Moons, L.M.; Kusters, J.G.; van Delft, J.H.; Kuipers, E.J.; Gottschalk, R.; Geldof, H.; Bode, W.A.; Stoof, J.; van Vliet, A.H.; Ketelslegers, H.B., et al. A pro-inflammatory genotype predisposes to barrett's esophagus. *Carcinogenesis* **2008**, *29*, 926-931.
65. Buas, M.F.; He, Q.; Johnson, L.G.; Onstad, L.; Levine, D.M.; Thrift, A.P.; Gharahkhani, P.; Palles, C.; Lagergren, J.; Fitzgerald, R.C., et al. Germline variation in inflammation-related pathways and risk of barrett's oesophagus and oesophageal adenocarcinoma. *Gut* **2017**, *66*, 1739–1747.
66. Yan, C.; Ji, Y.; Huang, T.; Yu, F.; Gao, Y.; Gu, Y.; Qi, Q.; Du, J.; Dai, J.; Ma, H., et al. An esophageal adenocarcinoma susceptibility locus at 9q22 also confers risk to esophageal squamous cell carcinoma by regulating the function of barx1. *Cancer Lett* **2018**, *421*, 103–111.
67. Abnet, C.C.; Freedman, N.D.; Hu, N.; Wang, Z.; Yu, K.; Shu, X.O.; Yuan, J.M.; Zheng, W.; Dawsey, S.M.; Dong, L.M., et al. A shared susceptibility locus in plce1 at 10q23 for gastric adenocarcinoma and esophageal squamous cell carcinoma. *Nat Genet* **2010**, *42*, 764–767.
68. Dura, P.; van Veen, E.M.; Salomon, J.; te Morsche, R.H.; Roelofs, H.M.; Kristinsson, J.O.; Wobbes, T.; Witteman, B.J.; Tan, A.C.; Drenth, J.P., et al. Barrett associated mhc and foxf1 variants also increase esophageal carcinoma risk. *Int J Cancer* **2013**, *133*, 1751–1755.

69. Wideroff, L.; Vaughan, T.L.; Farin, F.M.; Gammon, M.D.; Risch, H.; Stanford, J.L.; Chow, W.H. Gst, nat1, cyp1a1 polymorphisms and risk of esophageal and gastric adenocarcinomas. *Cancer Detect Prev* **2007**, *31*, 233–236.
70. Gross, R.E. *The Surgery of Infancy and Childhood: Its Principles and Techniques*; W.B. Saunders Co.: Philadelphia, PA, USA, 1953.
71. Lundell, L.R.; Dent, J.; Bennett, J.R.; Blum, A.L.; Armstrong, D.; Galmiche, J.P.; Johnson, F.; Hongo, M.; Richter, J.E.; Spechler, S.J., et al. Endoscopic assessment of oesophagitis: Clinical and functional correlates and further validation of the los angeles classification. *Gut* **1999**, *45*, 172–180.
72. Rasanen, K.; Itkonen, O.; Koistinen, H.; Stenman, U.H. Emerging roles of spink1 in cancer. *Clin Chem* **2016**, *62*, 449–457.
73. Jennings, R.E.; Berry, A.A.; Kirkwood-Wilson, R.; Roberts, N.A.; Hearn, T.; Salisbury, R.J.; Blaylock, J.; Piper Hanley, K.; Hanley, N.A. Development of the human pancreas from foregut to endocrine commitment. *Diabetes* **2013**, *62*, 3514–3522.
74. Gene expression for spink1 (ensg00000164266.10). Available online: https://gtexportal.org/home/gene/SPINK1 (accessed on 2021/10/15)
75. Alexander, D.H.; Lange, K. Enhancements to the admixture algorithm for individual ancestry estimation. *BMC Bioinformatics* **2011**, *12*, 246.
76. Wray, N.R.; Goddard, M.E.; Visscher, P.M. Prediction of individual genetic risk to disease from genome-wide association studies. *Genome Res* **2007**, *17*, 1520–1528.
77. Clc genomics workbench user manual. Available online: https://digitalinsights.qiagen.com/technical-support/manuals/ (accessed on 2021/10/15)
78. Bus, P.; Siersema, P.D.; Verbeek, R.E.; van Baal, J.W. Upregulation of mirna-143, -145, -192, and -194 in esophageal epithelial cells upon acidic bile salt stimulation. *Dis Esophagus* **2014**, *27*, 591–600.
